# Network analysis of the immune state of mice

**DOI:** 10.1038/s41598-021-83139-7

**Published:** 2021-02-22

**Authors:** Elohim Fonseca dos Reis, Mark Viney, Naoki Masuda

**Affiliations:** 1 Department of Mathematics, State University of New York at Buffalo, Buffalo, 14260 USA; 2 Department of Evolution, Ecology and Behaviour, University of Liverpool, Liverpool, L69 7ZB UK; 3 Computational and Data-Enabled Science and Engineering Program, State University of New York at Buffalo, Buffalo, 14260 USA; 4 Faculty of Science and Engineering, Waseda University, Tokyo, 169-8555 Japan

**Keywords:** Applied mathematics, Immunology

## Abstract

The mammalian immune system protects individuals from infection and disease. It is a complex system of interacting cells and molecules, which has been studied extensively to investigate its detailed function, principally using laboratory mice. Despite the complexity of the immune system, it is often analysed using a restricted set of immunological parameters. Here we have sought to generate a system-wide view of the murine immune response, which we have done by undertaking a network analysis of 120 immune measures. To date, there has only been limited network analyses of the immune system. Our network analysis identified a relatively low number of communities of immune measure nodes. Some of these communities recapitulate the well-known T helper 1 vs. T helper 2 cytokine polarisation (where ordination analyses failed to do so), which validates the utility of our approach. Other communities we detected show apparently novel juxtapositions of immune nodes. We suggest that the structure of these other communities might represent functional immunological units, which may require further empirical investigation. These results show the utility of network analysis in understanding the functioning of the mammalian immune system.

## Introduction

The vertebrate immune system defends animals against infection and disease. There has been extensive study of how the immune system functions, not only to understand its basic biology, but also to be able to manipulate it for therapeutic benefit. The immune system is a complex system with many cell populations, sub-populations and soluble molecules (such as cytokines) that act and interact to generate an immune response and to regulate that response. At its heart, the purpose of the immune system is to recognize foreign antigen molecules, to then remove, destroy or nullify them and their source, while usually retaining a molecular memory of the antigen in question. The immune system is dynamic, so that different components of the immune system respond depending on the type of antigen and its location in the individual. For example, immune responses to viruses infecting lung cells are qualitatively very different from the immune responses directed against macroscopic worms living in the gut^1^. Effector cells and molecules of the immune system act against invading pathogens, but these effects can also cause harm to the individual, so-called immunopathology^2^. Therefore, a tightly controlled, well-regulated immune system is needed to protect an individual from pathogen-induced harm, without causing direct harm to the individual in the process.

Despite the complex, multi-faceted nature of the immune system it is commonly analysed and quantified using relatively few immunological parameters; these parameters may be those that can feasibly be measured and / or those that are thought to be of particular importance with respect to what is being studied. This often low-dimension analysis contrasts with the complex, high-dimensionality of the immune response. To date there has been rather little large scale, immune system-wide analysis of the immune response. Therefore, within the study, analysis and understanding of the immune system there is a paradox: that immunology text books and review articles are filled with complex network diagrams (not unlike electrical circuits) showing how cells and molecules of the immune system are thought to act and interact^3–6^, but there has actually been limited immune system-level analyses of immunological data, which should actually underpin such system-level views of the immune system.

In the present work, we seek to address this by performing a network analysis of immunological data that we previously collected^7,8^. Networks (also called graphs) have proved to be a useful mathematical language to describe complex biological systems including interactions among cells, molecules and genes^9,10^. However, as far as we are aware, there are only a limited number of published studies that have used network analysis for investigating immunological data. Specifically, correlational networks have been constructed, similar to the present study, but limited to cytokine data only^11,12^, or a combination of data on cytokines, other soluble molecules and some cell populations, but from a small number of groups of subjects (i.e., a group of at most 11 participants)^13^. Other studies have undertaken a network analysis of components of the Nuclear Factor κB system, which is involved in mediating inflammation^14^, or networks of cytokines, chemokines and other relevant proteins in patients with pulmonary arterial hypertension^15^. A different approach that has been used is to construct networks based on protein abundance data, where the presence of those proteins in different immune cell populations was then analysed^16^. Network analysis has also been undertaken on proteomic data of haemopoietic cell types, to investigate interactions between those cell types^17^. Other analyses have constructed networks from prior immunological knowledge, and these networks have then been analysed, including by the addition of data^18,19^.

In contrast to these studies, here we use a large sample of real-world immunological data from hundreds of mice (*Mus musculus domesticus*), which has quantified a full range of serum proteins, cytokines, and cellular populations and sub-populations, totalling 120 immune measures. We build and analyse correlational networks from these data. Benefiting from the relatively large size of our network, we analyse the community structure of the immune networks aiming to reveal groups of immune responses and how they are interconnected^20^, which we show is not attainable by ordination-based analysis such as principal component analysis. In particular, we analyse signatures of T helper type 1 (Th1) and type 2 (Th2) responses in these networks. Crucially, our network analysis is purely data-driven and does not use a priori grouping of data based on biological knowledge. In this manner, we substantially advance network analyses of the immune system showing that the network recovers some known features of the immune response, thus showing the potential utility of such analyses in future immunological research.

## Materials and methods

### Data

**Figure 1:**
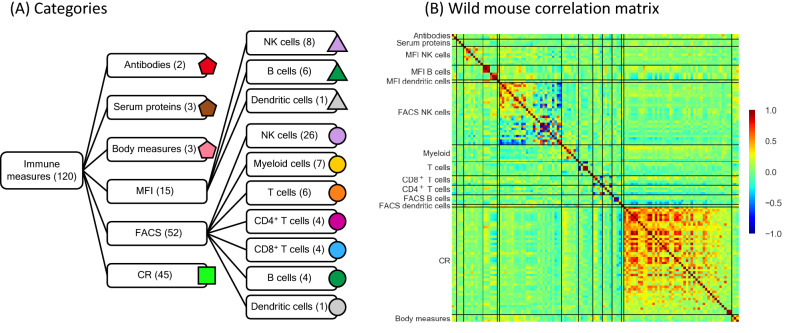
**(A)** Categories of immune measures. The numbers in the parentheses are the number of immune measures within that category. The symbol and colour associated with each category are used in the subsequent figures. The Mean Fluorescence Index (MFI) and Fluorescence Activated Cell Sorted (FACS) categories are divided into three and seven sub-categories, respectively. **(B)** Correlation matrix (coloured on a − 1 to + 1 scale) ordered according to the categories of immune measures for wild mice as shown in **(A)**. The solid black lines separate different categories of immune measures.

We used a previously published data set of the immune state of 460 wild mice (*Mus musculus domesticus*) and of 102 laboratory mice^7^. The wild mice were live trapped from 12 sites in the southern UK, and their mass, age, infection status and physiological state were measured, and these data are presented in our previous paper^7^. From the original 126 immune measures we pre-processed the data and excluded six immune measures (see Supplementary Text S1), leaving data for 120 immune measures, which are classified into six categories and sub-categories, as shown in Fig. 1A.

Concerning the six categories of immune measures (Fig. 1A) these are: (i) antibodies, which are the serum concentration of immunoglobulins G (IgG) and E (IgE); (ii) serum proteins, which are the concentration of the acute phase proteins serum amyloid P (SAP), Haptoglobin, and alpha-1 antitrypsin (AAT); (iii) spleen mass, number of spleen cells and body mass; (iv) the ex vivo activation state measured as mean fluorescence index (MFI) for three cell types, which has a total of 15 measures that are detailed in Supplementary Table S1; (v) fluorescence activated cell sorted (FACS)-derived percentages of seven cell types, which has a total of 52 measures, detailed in Supplementary Table S1, and (vi) cytokine responses (CR), which is the concentration of a cytokine produced in vitro after each of four types of stimulation, and one control, for nine cytokines, yielding 45 CR measures, detailed in Supplementary Table S1.

Many of the 120 immune measures had missing values, which is to be expected when working with wild populations. In particular the CR response data were missing for 223 wild mice (48%) and 75 laboratory mice (74%), and because of the central importance of cytokines in immune responses, we excluded these mice entirely from our analysis. For the remaining mice that had some missing values we imputed these, which we did by calculating the average value from the available observations, and then replaced the missing values by this calculated average. We carried out this imputation procedure separately for the wild and for the laboratory mouse data sets.

### Correlation of immune measures and comparative analyses

From these data, separately for the wild and for the laboratory mice, we calculated the Pearson correlation coefficient between each pair of immune measures, with each mouse being a sample. We used two methods to analyse these correlation matrices. Firstly, we built positive correlation networks using the 120 immune measures as network nodes. We placed an edge (which joins nodes) if, and only if, the correlation value exceeded a prescribed threshold; we explored the results at different correlation threshold values. Although using such a threshold discards the information contained in the value of the correlation coefficient, it does allow the application of various network analysis tools to such data. In fact, using thresholds with correlation networks have been shown to be useful for analysing various types of data such as neuroimaging^21,22^, genomic^23,24^, psychological^25^, climatological^26^ and financial^27^ data.

To build a connected network we used a threshold that was the largest possible correlation value at which the network remained connected and consisted of a single connected component. In these networks, the edge density is defined as $2e/N\left(N-1\right),$ i.e. the number of edges $e$ present in the network, divided by the maximum number of possible edges $N\left(N-1\right)/2$ in the network, with $N$ nodes. To detect communities of nodes in these networks we combined a stochastic block model (SBM)^28–31^ and a consensus clustering approach, uncovering the mesoscopic block structure of the correlation networks, which is described in Supplementary Text S2. Secondly, we used Principal Component Analyses (PCA), which is described in Supplementary Text S3.

## Results and discussion

### Pairwise correlations among wild mouse immune measures

The pairwise correlation matrix for the wild mice is shown in Fig. 1B, where the immune measures are grouped according to the categories of immune measures, and within each category individual measures are arranged in descending order of correlation coefficient. This shows that there is a concentration of large positive correlations among many members of the CR category. There are also strong positive and negative correlations within many of the other categories of immune measures (i.e., diagonal blocks in Fig. 1B). Within the FACS NK cells category there is a large proportion of immune measure pairs that are strongly, negatively correlated, while others are strongly, positively correlated. A frequency distribution of these pairwise correlation coefficients shows this mix of positive and negative correlations, and that there is a skew to positive correlation coefficients (Supplementary Fig. S1). While negative regulation is common in biological systems, in our network analyses, below, we focus on the positive correlations.

### Wild mouse immune network

**Figure 2:**
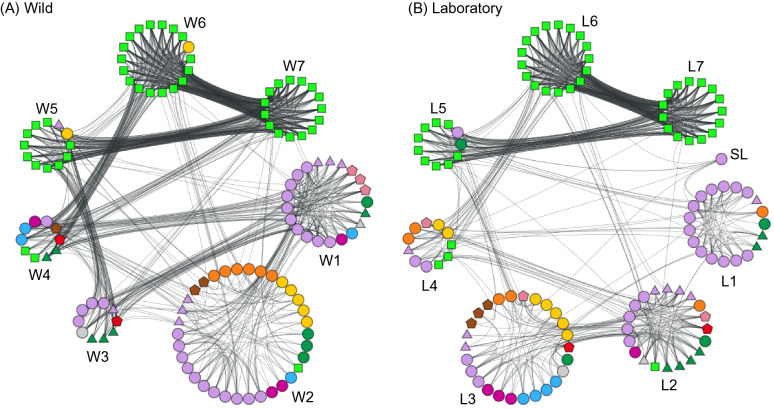
Community structure of the **(A)** wild and **(B)** laboratory mouse network, with the seven communities within each (W1–W7 and L1–L7), and the additional single (SL) node in the laboratory mouse network. Nodes in both networks are coded as Fig. 1A, and fully labelled networks are shown in Supplementary Fig. S2.

We constructed a wild mouse network with a threshold of 0.2, which was the highest correlation coefficient threshold that generated a connected network, consisting of seven communities and an edge density of 0.16. (Fig. 2A; Supplementary Fig. S2). Networks constructed with lower thresholds had a broadly similar structure of relatively few communities (specifically consisting of 10 or 12 communities, with a number of nodes not belonging to any community) and that the CR nodes were overwhelmingly concentrated in just 3 or 4 communities (Supplementary Fig. S3).

There are several notable features of the wild mouse network. Firstly, that the different categories of immune measures are distributed among the seven communities (Fig. 2A; Supplementary Fig. S2). Secondly, that three of the communities are almost exclusively composed of CR measures. Thirdly, that all communities are connected to each other (though to differing degrees), except for W3 and W6 that are not connected at all. Fourthly, that most communities have multiple within-community links as well as among-community links, though W3 and W4 have comparatively few within-community links.

These results show that the wild mouse immune network does not overtly resemble standard diagrams that summarise what is understood about the functioning of the immune system. This is notable because the network we present is wholly generated from analysis of empirical data. We suggest that the network and community structure revealed by this analysis represent functional aspects of the immune system. A priori we suggest that the different communities represent integrated, functional immunological units.

### Cytokines in the wild mouse network

To investigate whether the different communities in the network do represent functional immunological units we examined the network for evidence of known immunological features. Specifically, we sought evidence of cytokine communities consistent with the well-established Th1 vs. Th2 immune system polarization. Specifically, Interferon-gamma (IFN-γ) responses are indicative of Th1 responses, whereas Interleukin-4 (IL-4) and IL-13 responses are indicative of Th2 responses^1^. (IL-5 is similarly indicative of Th2 responses, but these data are not available in this study.) In the wild mouse network, the Th2-marker cytokine responses are present in communities W4 and W6, and the Th1-marker cytokine responses in W5 and W7. We tested whether the concentration of Th1/Th2-marker nodes into just two communities was statistically significant, by comparing their observed distribution with that for random assignments of these nodes into communities (Supplementary Text S4). In doing this we collapsed the five IL-13 nodes into one node, because each of these nodes was highly correlated with each other (Supplementary Text S4). The results show that the observed concentration of the Th2-marker nodes into W4 and W6 was significantly different from a random assignment of these nodes. The observed concentration of the Th1-marker nodes into W5 and W7 was marginally significantly different from a random assignment of these nodes. There are further features of the wild mouse network that also support W6 being a Th2 community, based on the strong links between W4 and W6 (Supplementary Figure S4). These are that W4 contains two of the five IL-4 cytokine responses, and that W4 contains two of the four CD8^+^ T cell populations, themselves indicative of Th2 responses (Supplementary Fig. S4). Overall, these results support the idea that there is polarization of Th1 vs. Th2 immune measures among different communities in the wild mouse network.

These results therefore show that this network analysis is able to recover evidence of known features of the mouse immune response. Therefore, the network approach may be a promising tool for analysing immunological data to reveal functional immunological groups. That our results have found other communities suggests that these may represent other, hitherto unknown, functional features of the immune response. Further observational and experimental work would now need to be undertaken to investigate this.

It is notable that the immunological phenotype of knock-out mice can be different from the predicted phenotype, which is in part due to the complex, inter-connected nature of the immune system, such that if a single feature is knocked-out, then there are multiple consequences throughout the immune system^32^. In an analogous fashion, the communities detected in this network analysis may represent immune-system wide immunological units, where their function or role (which, of course, may be multi-faceted) is not intuitively obvious. Further empirical investigation of these communities would be warranted.

### Wild mouse PCA analysis

**Figure 3:**
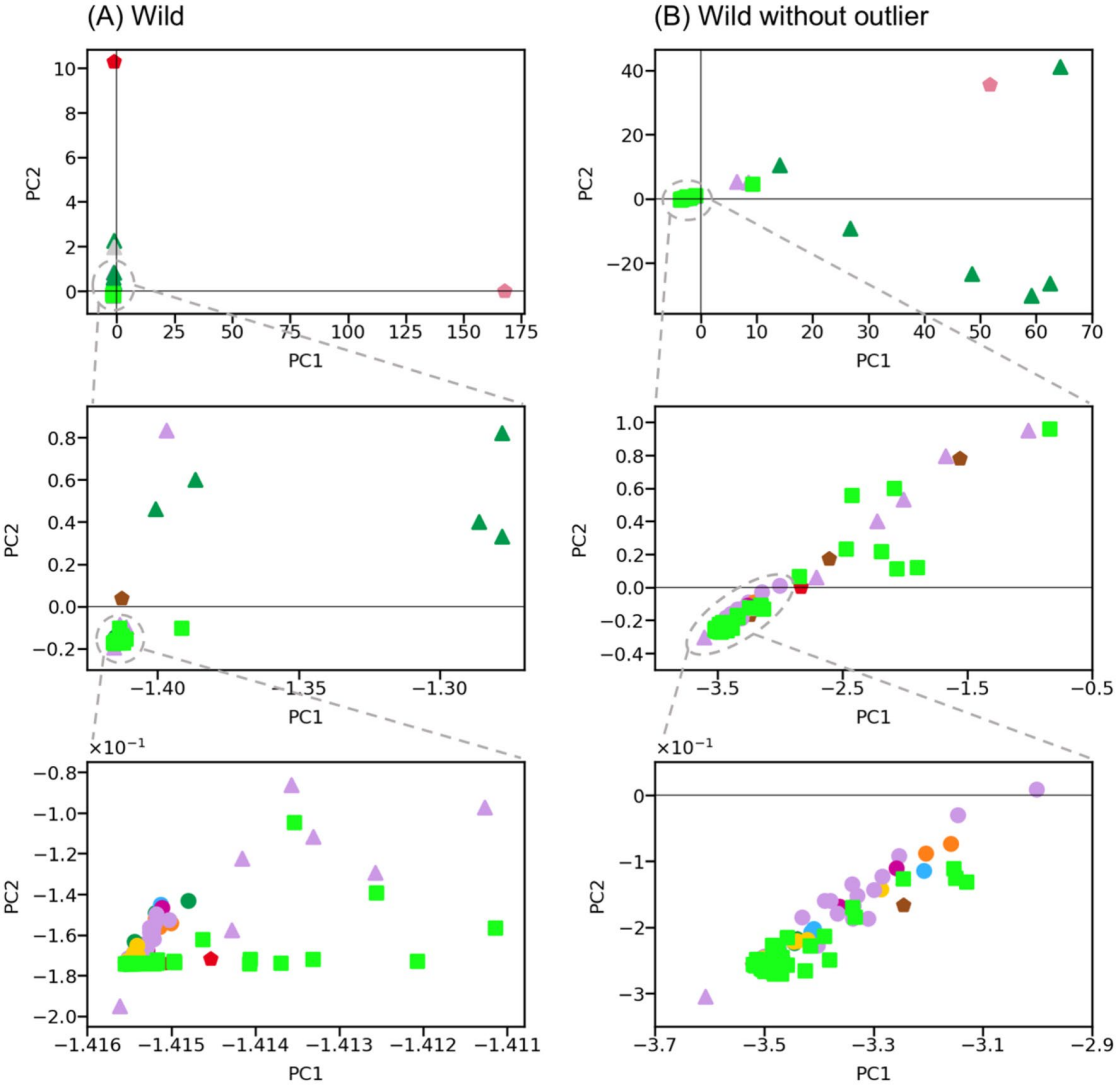
PCA results for the wild mouse data. Projection of the first (PC1) and the second (PC2) principal components of the immune measures **(A)** with and **(B)** without the outlier (number of spleen cells), with three levels of expansion (shown by the dotted lines) to reveal detail. Nodes are coded as in Fig. 1A.

To understand how network analysis compared with another method commonly used to analyse biological data, we used a principal component analysis (PCA) with the immune data. The first two principal components explained 99.6% and 0.4% of the variance in the data, respectively (Fig. 3A; Supplementary Text S3). The high concentration of the variance in the first component appears to be due to one outlier measure, the number of spleen cells. We therefore removed this measure and repeated the PCA with the remaining 119 measures. This showed that the first and second components now explained 67% and 19% of the variance, respectively (Fig. 3B). Notwithstanding, the PCA results do not show any clustering of the immune measures, except for MFI B cells. Similar results were obtained for the laboratory network (Supplementary Text S3).

Overall, this analysis did not reveal any strong patterns within the data, and nor did it recapitulate the different categories of immune measures present within our data set. This analysis also did not reveal the Th1 vs. Th2 cytokine communities, which were evident in the network analysis. We therefore conclude that network analysis of the present immunological data is superior to this PCA-based analysis in identifying functional immunological units.

### Comparison of wild and laboratory mouse immune networks

Our data set consisted of data obtained from wild mice and from laboratory mice. There are clear differences among mice living in these two environments, and immunological differences between these groups of mice have already been identified^7,8,33^. We therefore used network analysis to further compare the immune state of these two groups of mice, comparing single immune networks for both the wild (above) and laboratory mice. For the laboratory mouse network, the threshold was 0.42 and the edge density 0.13, and the network had seven communities and a single node that did not belong to any community (Fig. 2B; Supplementary Fig. S5). Laboratory mouse networks constructed with lower thresholds had a broadly similar structure of relatively few communities (specifically, consisting of 10 or 12 communities with some nodes not belonging to any communities) and with the CR nodes overwhelmingly concentrated in just 3 or 4 communities (Supplementary Fig. S3).

As for the wild mouse network, the different categories of immune measures are distributed among the communities; three communities are almost entirely composed of CR measures (as for the wild mouse network); many communities are connected to each other, though L1 and L6, L3 and L7, and L3 and L6 are not connected; and there are multiple within-community and among-community edges (as for the wild mouse network). Applying PCA to the laboratory mouse data showed that most of the data variance was encompassed in the first principal component (with the second principal component accounting for only 6 × 10^–6^ of the variance), and the analysis did not reveal any strong patterns, nor recapitulate features revealed by the network analysis (Supplementary Text S3; Supplementary Fig. S6).

In comparing the wild and laboratory immune networks, it is notable that both have three communities in which most CR nodes are concentrated (henceforth CR communities). Specifically, of the 45 CR nodes, 42 and 41 nodes belong to CR communities in the wild (W5, W6, W7) and laboratory (L5, L6, L7) mouse networks, respectively. The concentration of the CR nodes in these three communities was significantly higher than in a random assignment of the nodes among the seven communities, for both wild and laboratory networks (Supplementary Text S5). Moreover, the interconnections between the three CR communities is qualitatively the same for both networks—there is one CR community that is densely connected to the other two CR communities, which are in turn sparsely connected to each other. In other words, the three CR communities have a chain-like connection. In the wild network, community W7 has edge densities of 0.72 and 0.82 with communities W5 and W6, respectively, whereas the edge density between W5 and W6 is 0.06. Analogously, in the laboratory network, L7 has edge densities of 0.60 and 0.83 with L5 and L6, respectively, whereas the edge density between L5 and L6 is 0.03. These three CR communities have a significantly more chain-like connection than do randomly rewired CR subnetworks (Supplementary Text S6).

But, despite these structural similarities, the wild and laboratory mouse CR communities consist of different sets of nodes. We examined this further by computing the number of common nodes and the Jaccard index between each pair of communities in the wild and laboratory networks (Supplementary Text S7; Supplementary Fig. S7). Communities W7 and L7, which are strongly connected to the other two CR communities in the wild and laboratory networks, respectively, have only two CR nodes in common with a Jaccard index of 0.07 (Supplementary Fig. S7). In fact, this number (i.e., two) is not significantly different from the number of CR nodes in common when the nodes are randomly assigned to the three CR communities in the wild and laboratory networks (Supplementary Text S7). Moreover, the number of common nodes between W5 and W6 and L5 and L6 in all pair-wise W to L comparisons is not different from the number in randomized networks (Supplementary Text S7). Therefore, the nodes in the CR communities W5 and W6 (which are both strongly connected to W7, but not strongly connected to each other) are not concentrated in either L5 or L6 (which are both strongly connected to L7). Rather, many nodes in W6 and W7 are found in L7 and L6, respectively (Supplementary Text S7). For example, in the wild network all of the IL-1*β*, IL-12p70, and IL-13 nodes are each within one community (*i.e*., W7, W6, and W6, respectively), whereas in the laboratory network only the IL-12p70 nodes are all within one community (*i.e*., L7) (Supplementary Table S2). In summary, although the mesoscale structure of the CR communities is similar between the wild and laboratory mouse networks, the composition of each CR community is substantially different between the two networks. This difference might represent functional immunological differences between the cytokines of the wild and laboratory mice.

We also compared the immune networks from the perspective of the Th1 vs. Th2 cytokines, finding that the laboratory mouse network differs from that of the wild mice. Specifically, the IFN-γ Th1-marker cytokine nodes are in L2, L5 and L6, and the Th2-marker cytokine nodes are in L5, L6 and L7. We find that the Th1 or Th2 cytokine nodes in the laboratory network are not more concentrated than in a random assignment of the nodes into communities (Supplementary Text S4). We suggest that this difference between wild and laboratory mice represents a functional immunological difference between such mice. Thus, in wild mice extensive antigen exposure has stimulated both Th1 and Th2 immune responses that, with their cross-regulation, are manifest as separate communities within the immune network. In contrast, the laboratory mice will have had very limited antigenic exposure such that meaningful Th1 vs. Th2 immune responses have not been generated, hence their absence from the immune network.

We also investigated the distribution of 12 humoral response nodes, consisting of antibody concentration, and FACS and MFI of B cells (Fig. 1). We found that six of these are in L2 and three in L1 (Fig. 2), which is suggestive of some community concentration of these humoral nodes. This pattern contrasts with the wild network, where these 12 nodes were in communities W1-4. These nodes are significantly more concentrated in these laboratory network communities compared with random assignment of these nodes; in the wild network the observed distribution of these nodes was not different from a random assignment (Supplementary Text S8). A different arrangement of these humoral response nodes between the wild and laboratory mouse networks may be due to their different humoral responses, with wild mice having very high concentrations of antibodies compared with laboratory mice^7^.

There are other similarities and differences among the wild and laboratory mouse networks. In both, a majority of NK cell nodes (i.e. FACS and MFI nodes) belong to two communities, W1 and W2, and L1 and L2. Communities W1 and L1 are predominately composed of NK cell nodes and all the eight nodes that they share are FACS NK cell nodes. Among the seven nodes shared by communities W2 and L2, five are NK cell nodes (three FACS NK cells and two MFI NK cells). In both networks, the edges within each of these two NK cell communities are more dense than between them. Consistent with this observation, the pairwise correlations between nodes within L1 and L2 are mostly strongly positive whereas those between L1 and L2 are mostly strongly negative (Supplementary Fig. S8). This result may imply negative biological regulation among NK cell nodes.

T cell nodes differ among the wild and laboratory mouse networks. In the wild network, T cells are confined to a single community, W2, whereas they are dispersed among four communities in the laboratory mouse network (Fig. 2; Supplementary Figs. S2 and S5). This result is likely due to greater functional interaction among these cells in wild, antigen-experienced mice, compared with laboratory mice^33^.

There are therefore some similarities among the wild and laboratory networks, but this is not completely unexpected since both wild and laboratory mice are the same species, and so have the same immune system with the same components, which will necessarily have some, even substantial, shared function. Despite the mesoscale similarity, the node composition of the individual communities differs substantially between the wild and laboratory networks. We find considerable differences between the two networks in: the identity of nodes constituting the three CR communities; evidence for separation of Th1 vs. Th2 cytokine nodes; and different arrangement of the T cell nodes. However, when we compare the overall community structure of the two networks, i.e., how all the nodes are partitioned into communities, we were not able to find statistical support for differences in the structure of the wild and laboratory mouse networks (Supplementary Text S9; Supplementary Table S5).

### Negative correlations

We have not considered negative pairwise correlations because the thresholds for creating the networks from the correlation matrices were positive. However, mindful that in biological systems negative regulation is common, we also briefly analysed how the immune measures are connected through negative correlations. In these negative networks, node pairs that are strongly negatively correlated were assumed to form the edges. The negative networks have substantially more communities (9 and 17 for the wild and laboratory, respectively) than the positive networks such that their community structure is more difficult to interpret (Supplementary Fig. S9). However, the CR nodes are predominately grouped together within several communities in both negative networks, similarly to the positive networks. Specifically, in the wild mouse negative network, 43 out of the 45 CR nodes are in one of two communities; for the laboratory mouse negative network 39 CR nodes belong to one of the four communities. Because CR nodes tend to be strongly positively correlated with each other, there is no direct connection between pairs of CR nodes in the negative networks. However, in the SBM, a pair of nodes tend to belong to the same community if they have similar patterns of connectivity to the other nodes in the network. This is the case even if the direct connectivity between the two nodes is not strong, or even absent. This property of the SBM explains why various CR nodes belong to the same community despite the absence of the direct connections among them. The results for the negative networks suggest that, for both wild and laboratory mice, the groups of CR nodes have relatively similar patterns of negative connectivity towards other CR or non-CR nodes.

## Conclusion

We present a network analysis of the immune state of mice based on a large, comprehensive set of immune measures and a large sample of mice. This analysis therefore considerably expands the previous use of network analysis for investigating the immune system and immune responses. Our analysis is able to recover some known features of immune function (the Th1 vs. Th2 cytokine polarisation) suggesting, by extension, that the other communities that we have identified may also represent immunological functional units. We show that the network analysis is superior to PCA for our data. However, formal statistical analysis failed to identify differences between the wild and laboratory mouse networks. We suggest that network analyses such as the one we have presented complement other non-network methods of analysis, by providing measures not available from other methods.

The present study has some limitations. First, we necessarily discarded a considerable portion of data during our pre-processing steps due to missing values. Studies of wild animals will routinely confront such limitations. Therefore, the pre-processing of data that we have undertaken may provide a model for how this matter can be addressed in similar analyses of analogous data of other populations. A larger data set would significantly enhance the precision of the present findings. Second, we were not able to find statistical support for differences between the wild and laboratory mouse networks. As above, this may be due to the relatively small size of the data set, compared to the number of nodes. Sampling wild mice and making these multiple immune measures is a substantial piece of work and we note that this data set is the largest existing immunological data set for wild rodents. Third, we applied a threshold to the correlation matrices to create the correlation networks. This procedure loses potentially useful information contained in the value of the correlation. The choice of the threshold values we used was not determined a priori, but rather determined functionally by using the highest value that resulted in a single connected component^34,35^. Alternative options to select the threshold value could be based on an optimization method^35^ or the use of network quantities directly applied to correlation matrices^36–38^. Fourth, we have constructed networks based on either positive or negative correlations, which therefore does not allow a network based on correlations that are in different directions. Biological systems are typically both positively and negatively regulated, and so a network-style analysis that would allow both positive and negative correlations could be more valuable.

The network analysis of wild mouse populations presented here is a novel analysis of a unique data set. It generates a holistic, data-driven view of the mammalian immune system and the dynamics of its function as the animals themselves interact with diverse and dynamic environments. Humans are, arguably, immunologically more akin to wild mice than to laboratory mice^7,8,39^, and the diversity of immune state among people could also be analysed using network analyses. Such an approach may usefully be used to better understand the mammalian immune system and its function in protecting individuals from infection and disease.

## Supplementary Information

Supplementary Information.
