# Supplementary material for: Network analysis of the immune state of mice
Source: Sci Rep. 2021 Feb 22;11:4306. doi: 10.1038/s41598-021-83139-7 (PMC7900184; doi:10.1038/s41598-021-83139-7)
Supplement: Supplementary file 1 — Supplementary Information. [file 41598_2021_83139_MOESM1_ESM.docx]

**Supplementary Information**

**Network Analysis of the Immune State of Mice**

**Elohim Fonseca dos Reis^1^, Mark Viney^2^ and Naoki Masuda^1,3*^**

^1^ Department of Mathematics, State University of New York at Buffalo, Buffalo, United States

^2^ Department of Evolution, Ecology and Behaviour, University of Liverpool, Liverpool, L69 7ZB, United Kingdom

^3^ Computational and Data-Enabled Science and Engineering Program, State University of New York at Buffalo, Buffalo, United States

*email: naokimas@buffalo.edu

**Supplementary Text S1. Data pre-processing**

We pre-processed the data as follows. The concentrations of cytokines were measured using Bioplex Pro kits (M60-009RDPD & MD0-00000EL, Bio-Rad, UK). Therefore, the range of cytokine concentrations for which data were robust is defined by empirically derived standard curves. When the observed concentration of a cytokine fell outside the standard range of these assays [1], the readings were classified as out of range (OOR), being either below (“OOR*<*”) or above (“OOR*>*”) the standard range. We set “OOR *<*” measures to 0.001 [1], and treated “OOR *>*” as a missing value. We removed the age and IgA measures [1] because they were absent for all the laboratory mice. In the FACS category we removed the %D+G2Hi+G2Lo+H+, %D+G2Hi+G2Lo+H-, %D-G2Hi+G2Lo+H+ and %D-G2Hi+G2Lo+H- measures because they were absent for all wild and laboratory mice. As a result of removing these six measures, we had a final total of 120 immune measures.

**Supplementary Text S2. Network community detection**

In the microcanonical formulation of the stochastic block model (SBM) that we used, one minimizes the description length of the network [2], such that one partitions the nodes in the network $\mathcal{G}$ into $B$ blocks to find $b=\{b_{1},\ldots,b_{N}\}$, where $b_{i} \in\{1,2,\ldots,B\}$ is the group membership of node $i$. The probability of generating the observed network $\mathcal{G}$ given partition $b$ is denoted by $P\left( \mathcal{G} | \theta,b \right)$, where *θ* is the set of the additional parameters that control the connectivity between blocks. The probability that the observed network $\mathcal{G}$ is generated by a partition $\boldsymbol{b}$ is given by the following Bayesian posterior probability:

$$\begin{aligned} P\left( \boldsymbol{b} | \mathcal{G} \right)=\frac{P\left( \mathcal{G} | \theta,\boldsymbol{b} \right)P\left( \theta,\boldsymbol{b} \right)}{P\left( \mathcal{G} \right)}.\#\left( S1 \right) \end{aligned}$$

The numerator of Eq. (S1) can be written as $P\left( \mathcal{G} | \theta,\boldsymbol{b} \right)P\left( \theta,\boldsymbol{b} \right)=\exp\left( -H \right)$, where

$$\begin{aligned} H=-\ln P\left( \mathcal{G} | \theta,\boldsymbol{b} \right)-\ln P\left( \theta,\boldsymbol{b} \right)\#\left( S2 \right) \end{aligned}$$

is the description length of network G. Maximizing Eq. (S1) is equivalent to minimizing the description length. We used the non-degree corrected version of the SBM algorithm because it yielded smaller description lengths. Specifically, after 10^3^ minimizing attempts, the average and standard deviation of the description length for the corrected and the non-corrected version was equal to 2575±3 and 2526±4, respectively, for the wild mice and 2118±5 and 2047±5, respectively, for the laboratory mice.

To infer the best partition given by the SBM, we employed a Markov Chain Monte Carlo (MCMC) algorithm [3] using the graph-tool library [4]. We start from an initial partition $b_{0}$, which is obtained from an agglomerative heuristic method [3]. Then, in each step, we propose a move by selecting a node $i$ and choosing a new tentative group membership $b_{i}^{'}$ for node $i$ with a specific probability that imposes no bias and preserves the ergodicity [3]. The proposed move $b\to b^{'}$, where $b_{j}^{'}=b_{j}$ for all $j \neq i$, is accepted or rejected according to the Metropolis-Hastings’ criterion [5, 6]. This procedure preserves the detailed balance and minimizes the final description length. Each attempt to move a node is applied sequentially, such that nodes are visited one by one in a random order.

When all the *N* nodes have attempted to move once, we say that a sweep has been completed. Because the method is stochastic, a different block structure may appear in each run of the algorithm. To cope with this stochasticity, we implemented the consensus clustering procedure composed of the following three phases. In the first phase, we identify the most probable number of communities of the network. To this end, we ran the algorithm for 10^2^ different initial partitions of the nodes. Then, for each initial condition, we carried out MCMC sweeps and recorded the maximum and minimum values of the description length across sweeps. The node partitions were accepted as being stable if 10^3^ sweeps were completed with less than two record breakings of the description length, where a record breaking is defined by the appearance of the description length value that is larger than the largest value among all the previous sweeps. For each of the 10^2^ initial conditions, we recorded, at an interval of 10^2^ MCMC sweeps, the number of communities 10^4^ times. Then, for each initial condition, we selected the number of communities that appeared the most times in the 10^4^ observations. Finally, we determined the number of communities of the network as the most probable number of communities among the 10^2^ initial conditions. In the second phase, we fixed the number of communities to the value determined in the first phase and inferred the probability that each node belongs to each community as follows. For each of another 10^2^ different initial partitions of nodes, we underwent the same transient period of MCMC sweeps as in the first phase. Then, for each of the 10^2^ initial conditions, we collected 10^5^ configurations at an interval of 10^2^ MCMC sweeps and calculated the fraction of the 10^5^ configurations in which each node belonged to each community. Finally, for each initial condition, we defined the community of each node as the community to which the node belonged the most times. Therefore, at the end of the second phase, we had 10^2^ node partitions. In the third phase, we performed a consensus clustering procedure. To this end, we counted the number of times that each pair of nodes belonged to the same community among the 10^2^ node partitions found in the second phase. We then concluded that any pair of nodes belonged to the same community if they did so in at least 90% of the 10^2^ node partitions. If a node did not belong to the same community with any other node in at least 90% of the 10^2^ node partitions, then this node was judged to form a single-node community.

**Supplementary Text S3. PCA methods and results for the laboratory mouse data**

We used principal component analysis (PCA) to embed the immune measures in a lower dimensional space and to observe how the immune measures were statistically grouped. Because the various immune measures have different units of measurement and magnitudes, we linearly normalized each immune measure to have a mean of 0 and a standard deviation of 1 before carrying out the PCA. We performed this scaling separately for the wild and laboratory mice. For the wild mice, the fraction of the variance explained by the first and second principal components was 0.996 and 0.004, respectively (**Fig. 3A**). We observe that a single measure, the number of spleen cells (shown as a pink pentagon in **Fig. 3A**), is an outlier. This outlier causes a majority of the other immune measures to collapse close to each other in the PCA space. Therefore, we removed this immune measure and repeated the PCA analysis on the remaining 119 immune measures (**Fig. 3B**). In this case, the variance explained by the first and second components for the wild mice was 0.67 and 0.19, respectively.

The corresponding results for the laboratory mice are shown in **Supplementary Fig. S6**. When all immune measures were included, the first and second principal components explained almost 1 and 6×10^−6^ of the variance, respectively (**Supplementary Fig. S6A**). As for the wild mice, the number of spleen cells appeared to be an outlier causing the large proportion of the variance to be explained by the first component. Therefore, we removed this immune measure and repeated the PCA on the remaining 119 immune measures (**Supplementary Fig. S6B**). In this case, the variance explained by the first and second components was 0.77 and 0.12, respectively.

Therefore, for both wild and laboratory mice, the first two principal components together explained a large proportion of the variance of the data, even with the removal of the number of spleen cells. Regardless of whether or not we removed the number of spleen cells, the PCA results suggest that there is no obvious grouping of the immune measures for either the wild or laboratory mice. Almost all measures are closely grouped around the origin (**Fig. 3**; **Supplementary Fig. S6**, top panels). Magnification of the plots near the origin reveals for both wild and laboratory mice some separation of B cell activation states (shown as green triangles) from the other immune measures (**Fig. 3**; **Supplementary Fig. S6**, middle panels). This PCA result still does not show notable clustering of immune measures except for measures of B cell activation. A second level of magnification showed some separation of the cytokine measures (shown as green squares) from the rest of the measures (**Fig. 3**; **Supplementary Fig. S6**, bottom panels). However, overall the PCA results did not show a clear evidence of clustering among the immune measures.

**Supplementary Text S4. Th1 *vs*. Th2 polarization**

The well-established Th1 *vs*. Th2 immune system polarization can be observed in the wild network but not in the laboratory network (**Supplementary Table S2**). In the wild network, the five nodes of the IFN-$\gamma$ Th1-marker cytokine are concentrated in two communities, W5 and W7, and the five nodes of each of the IL-4 and IL-13 Th2-marker cytokines are concentrated in communities W4 and W6. This degree of concentration is not present in the laboratory network; the five Th1-marker cytokine nodes are in communities L2, L5, and L6, and the ten Th2-marker cytokine nodes are in L5, L6, and L7.

Before statistically testing the distribution of these nodes we addressed the possibility that the five stimulation conditions for each cytokine (*i.e*., IFN-$\gamma$, IL-4, IL-13) were pseudoreplicates, and so we asked whether or not the Th1-marker cytokine nodes were sufficiently independent from each other, and whether or not the Th2-marker cytokine nodes were sufficiently independent from each other. For example, if the five stimulation conditions for IFN-$\gamma$ were not sufficiently independent, it would be unsurprising that the five the IFN-$\gamma$ nodes belonged to a single community. In this scenario then the five nodes should be better considered as one supernode. To address this we calculated the average and standard deviation of the correlation coefficients between each pair of the Th1-marker cytokine nodes and between each pair of Th-2 marker cytokine nodes, separately for the wild and laboratory mice.

For the wild mice, the correlation coefficient between the IFN-$\gamma$ nodes, between the IL-4 nodes, and between the IL-13 nodes was 0.36 $\pm$ 0.19 (mean $\pm$ standard deviation), 0.35 $\pm$ 0.25, and 0.69 $\pm$ 0.21, respectively. The correlation coefficient between an IL-4 node and an IL-13 node was 0.44 $\pm$ 0.24. For the cytokine nodes that are not Th1 or Th2-marker cytokines, the correlation coefficient was 0.35 $\pm$ 0.22.

Based on these results, we concluded that the IL-13 nodes had to be considered as one supernode because they were highly correlated with each other, and correlated considerably more strongly than the other cytokine node pairs. For the other cytokines, based on these results, we do not consider that they should be treated as supernodes. Therefore, we consider that there were effectively six Th2-marker cytokine nodes (*i.e.*, five IL-4 nodes and one IL-13 supernode) that were distributed in two communities of the wild network. Because all the five IL-13 nodes were in community W6, we subtracted four nodes from the number of nodes of community W6 in the following statistical analysis.

For the laboratory mice, the correlation coefficient between the IFN-$\gamma$ nodes, between the IL-4 nodes, between the IL-13 nodes, and between the IL-4 and IL-13 nodes was 0.33 $\pm$ 0.39, 0.60 $\pm$ 0.29, 0.71 $\pm$ 0.24, and 0.66 $\pm$ 0.28, respectively. For pairs of cytokine nodes excluding the Th1 and Th2-marker nodes the correlation was 0.47 $\pm$ 0.30. These results indicate that, as in the case of the wild network, the IL-13 nodes were correlated with each other considerably more strongly than were the other cytokine node pairs. Therefore, we treated the five IL-13 nodes as one supernode. Of the five IL-13 nodes, three were in L7, one in L5, and one in L6, and so we subtracted one node from L5, one node from L6, two nodes from L7, and kept one node in L7 in the following statistical analysis.

To investigate statistically the arrangement of the Th1 and Th2 nodes among communities in the networks, we defined $Y_{1}$ as the number of communities to which Th1 nodes belonged and $Y_{2}$ as the number of communities to which Th2 nodes belonged. In the original wild network, $Y_{1}=2$ and $Y_{2}=2$. In the original laboratory network, $Y_{1}=3$ and $Y_{2}=3$. Then, separately for the wild and laboratory networks, we uniformly randomly (*i.e.*, without bias) assigned each node to each of the seven communities keeping the number of nodes in each community the same as in the community structure of the original network. In other words, after the random assignment, each node belongs to an arbitrary community with the probability of being assigned to that community proportional to the size of the community (*i.e.*, the number of nodes that the community contains in the original network). Note that we reduced the number of IL-13 nodes from five to one, as described above. We excluded the single-node community in the case of the laboratory network. We generated 10^5^ random assignments. For each of the 10^5^ assignments, we computed $Y_{1}$ and $Y_{2}$. We calculated the average and standard deviation of $Y_{1}$ and $Y_{2}$ for the 10^5^ assignments and then the Z score of $Y_{1}$ and $Y_{2}$ for the original network. The Z score for $Y_{1}$, for example, is given by $Z = \left( Y_{1} - \mu_{\text{rand}} \right)/\sigma_{\text{rand}}$, where $\mu_{\text{rand}}$ and $\sigma_{\text{rand}}$ are the average and standard deviation of $Y_{1}$ over the 10^5^ random assignments, respectively.

The average and standard deviation of $Y_{1}$ and $Y_{2}$ for the random assignments was 3.62 $\pm$ 0.78 and 4.05 $\pm$ 0.84 for the wild network, respectively. The corresponding Z score for $Y_{1}$ and $Y_{2}$ was equal to −2.07 (*p* = 0.038, uncorrected; *p* = 0.077, Bonferroni corrected with multiplicity two) and −2.43 (*p* = 0.030, Bonferroni corrected), respectively. Therefore, in the wild network the observed concentration of the Th2 nodes in a small number of communities is significantly different from their random assignment, and that of the Th1 nodes is marginally significant (*i.e.*, significant only in the absence of the multiple comparison correction). For the laboratory network, the average and standard deviation of $Y_{1}$ and $Y_{2}$ for the random assignments was 3.70 $\pm$ 0.76 and 4.15 $\pm$ 0.82, respectively. The Z score for $Y_{1}$ and $Y_{2}$ was equal to −0.92 (*p* = 0.36, uncorrected) and −1.40 (*p* = 0.16, uncorrected). Therefore, for the laboratory mouse network the arrangement of the Th1 or Th2 nodes in a small number of communities is not significantly different from their random assignment.

**Supplementary Text S5. CR communities**

In both wild and laboratory networks, there are three communities that are almost exclusively composed of CR nodes, which we call CR communities. We statistically tested if indeed there were just three communities that contain a large number of CR nodes. To do this we, first, calculated the proportion of CR nodes present in each community separately for the wild and the laboratory networks. We excluded the single-node community in the case of the laboratory network. We denote the proportion of CR nodes in the *i*th community by $f_{i}$, where *i* = 1, ..., 7. If our hypothesis holds true, then $f_{i}$ for three communities should be large and $f_{i}$ for the other four communities should be small. To test our hypothesis we, secondly, focused on the third and fourth largest values of $f_{i}$ among $f_{1}{, ...,f}_{7}$, which we denote by $f_{\mathrm{third}}$ and $f_{\mathrm{fourth}}$, respectively. If the CR nodes are concentrated in the top three communities, $f_{\mathrm{third}}$ should be large, and $f_{\mathrm{fourth}}$ should be small. Note that, by definition, three out of the seven communities have $f_{i}$ values that are greater than or equal to $f_{\mathrm{third}}$ and the other four communities have $f_{i}$ values that are less than or equal to $f_{\mathrm{fourth}}$. In the wild network $f_{\mathrm{third}}$ = 0.22 and $f_{\mathrm{fourth}}$ = 0.04; in the laboratory network $f_{\mathrm{third}}$ = 0.20 and $f_{\mathrm{fourth}}$ = 0.07. Third, we uniformly randomly assigned each node to each of the seven communities keeping the number of nodes in each community the same as in the community structure of the original network. We generated 10^3^ random assignments. Fourth, we calculated $f_{\mathrm{third}}$ and $f_{\mathrm{fourth}}$ for each of the 10^3^ random assignments. Fifth, we used $f_{\mathrm{third}}/f_{\mathrm{fourth}}$ as the statistic to test our hypothesis; $f_{\mathrm{third}}/f_{\mathrm{fourth}}$ should be large if the CR nodes are concentrated in three communities. Sixth, we calculated the average and standard deviation of $f_{\mathrm{third}}/f_{\mathrm{fourth}}$ for the 10^3^ random assignments and then the Z score for the original wild or laboratory network.

The Z score for $f_{\mathrm{third}}/f_{\mathrm{fourth}}$ in the wild and laboratory networks was 14.6 and 7.1, respectively. These Z score values were significantly larger than in the randomly assigned case (Z = 7.1 corresponds to *p* = 2.5 x 10^−12^, Bonferroni corrected with multiplicity two). Therefore, the observed concentration of the CR nodes in the three communities is statistically supported for both wild and laboratory networks.

**Supplementary Text S6. Connectivity among the CR communities**

The three CR communities appear to show a chain-like interconnection in both wild and laboratory networks (**Supplementary Figs. S2, S5 and S8**). Specifically, it appears that one of the three CR communities is densely connected to both of the other CR communities, but that these communities are themselves sparsely connected to each other. In the wild network, community W7 is densely connected to W5 and W6. In the laboratory network, community L7 is densely connected to L5 and L6. To statistically test whether or not the three CR communities are connected in a chain-like manner, we considered a subnetwork consisting of only the three CR communities, which we call the CR subnetwork. Then, separately for the wild and laboratory networks, we calculated a measure, denoted by $X$, as follows. We first calculated the edge density between each pair of CR communities. For example, the edge density between W5 and W6 is the number of edges between W5 and W6 divided by the product of the number of nodes in W5 and in W6. The edge density ranges between 0 and 1. Then, we defined $X$ as the sum of the two largest values of the edge densities between the CR communities less the smallest value. For example, in the case of the wild network, we obtain

| $X$ | $= \left( \text{edge density between W5 and W7} \right)+\left( \text{edge density between W6 and W7} \right)$ |  |  |
| --- | --- | --- | --- |
|  | $-\left( \text{edge density between W5 and W6} \right).$ | (S3) | |

A chain-like structure consisting of three communities yields a large $X$ value. Next, using the configuration model [11], we randomized CR subnetworks by rewiring the edges but preserving the degree (*i.e.*, defined as the number of edges connected to the node) of each node. We also assumed that the randomization did not change the community to which each node belongs. We generated 10^3^ randomized CR subnetworks and then calculated the average and standard deviation of $X$ over the 10^3^ randomized CR subnetworks.

The $X$ value for the original and randomized wild networks was 1.48 and 1.10 ± 0.03 (mean ± standard deviation), respectively, and 1.40 and 1.10 ± 0.03 for the original and randomized laboratory networks, respectively. The Z score for $X$ in the wild and laboratory networks was equal to 12.0 and 8.4, respectively. In both cases, the Z score is significantly larger than the randomized CR subnetworks (Z = 8.4 corresponds to *p* = 8.9 x 10^−17^, Bonferroni corrected with multiplicity two). This result supports the conclusion that the three CR communities have a chain-like structure in both the wild and laboratory networks.

**Supplementary Text S7. Comparison of individual network communities**

We compared the community structure of different immune networks in two ways. Firstly, by counting the number of nodes shared by the two communities. Second, by computing the Jaccard index $J$ between the two communities, defined by

$$\begin{aligned} J\left( A,B \right)=\frac{\left| A\cap B \right|}{\left| A\cup B \right|}=\frac{\left| A\cap B \right|}{\left| A \right|+\left| B \right|-\left| A\cap B \right|},\#\left( S4 \right) \end{aligned}$$

where $A$ and $B$ are the set of nodes in the two communities being compared, and $\left| A \right|$, for example, is the number of nodes in $A$. The number of common nodes and Jaccard index between the wild and laboratory communities are shown in **Supplementary Figs. S7A** and **S7B**, respectively.

To examine whether or not the observed number of common nodes between a CR community in the wild network and in the laboratory network was different to the number present when nodes were randomly assigned into communities, we performed a statistical test as follows. First, we uniformly randomly shuffled the nodes in W5, W6, and W7 to create new communities, denoted by W5’, W6’, and W7’. The communities W5’, W6’, and W7’ have the same number of nodes as W5, W6, and W7, respectively. After the random shuffling, each node belongs to one of the three new communities with a probability that is proportional to the size of the community (*i.e.*, number of nodes in the community). We also performed this process for L5, L6, and L7, creating new communities, L5’, L6’, and L7’. Second, we computed the number of common nodes and the Jaccard index between a CR community in the randomly shuffled wild network (*i.e.*, W5’, W6’, or W7’) and a CR community in the randomly shuffled laboratory network (*i.e.*, L5’, L6’, or L7’). Third, we calculated the average and the standard deviation of the number of common nodes and the Jaccard index over 10^5^ such randomizations. Fourth, we calculated the Z score of the number of common nodes and the Jaccard index.

The average and standard deviation of the number of common nodes and the Jaccard index are shown in **Supplementary Table S3**. The corresponding Z scores are shown in **Supplementary Table S4**. Overall, this shows qualitatively similar results for the Z scores of the number of common nodes and of the Jaccard index. Communities W7 and L7 share two nodes. We find that this number (*i.e.*, 2) is not significantly different from the number of nodes that these two communities share when the wild and laboratory networks are randomized (Z = −1.62 and Z = −1.50 for the number of common nodes and the Jaccard index, respectively; these Z values correspond to *p* = 0.11 and *p* = 0.13, uncorrected; **Supplementary Table S4**). Similarly, the number of nodes that W5 and L5 share (*i.e.*, 1**; Supplementary Fig. S7**); that W5 and L6 share (*i.e.*, 6); that W6 and L5 share (*i.e.*, 4) and that W6 and L6 share (*i.e.*, 1) are not significantly larger than that expected for the randomized network (**Supplementary Table S4**). Therefore, we conclude that the nodes in W5 and W6 (which are both strongly connected to W7 but not strongly connected to each other) are not significantly more concentrated in L5 or L6 (which are both strongly connected to L7 but not between each other). Note that a significantly negative Z score (without correction for multiple comparison) for the W6-L6 pair indicates that W6 and L6 nodes share less nodes than expected from the randomized network. Rather, W7 and L6 share many nodes (*i.e.*, 9 nodes) and W6 and L7 share many nodes (*i.e.*, 12 nodes); both of these numbers are significantly larger than those for the randomized networks (Z = 4.26 and Z = 2.57 for the W6-L7 and W7-L6 pair, respectively; these Z values correspond to *p* = 4.1 x 10^−5^ and *p* = 0.020, Bonferroni corrected with multiplicity two; the results are more significant with the Jaccard index).

**Supplementary Text S8. Concentration of humoral response nodes**

We investigated whether there was a concentration of humoral response (HR) immune measure nodes in communities in the laboratory and wild networks. We focused on 12 HR nodes, consisting of antibody concentration and FACS and MFI of B cells (**Fig. 1**). In the laboratory network, six of the HR nodes are in L2 and three are in L1 (**Fig. 2**). Such a concentration is not apparent in the wild network, where the HR nodes spread across W1, W2, W3, and W4. We statistically asked if this distribution of HR nodes among communities was different from the randomized data as follows. First, separately for the wild and laboratory networks, we calculated the fraction of HR nodes in each community. We denoted by $f_{i}^{'}$ the fraction of HR nodes in community $i$, where $i=1, \ldots, 7$. We excluded the single-node community in the laboratory network. Second, we calculated the standard deviation of ${f^{'}}_{1},\ldots,{f^{'}}_{7}$, which we denoted by $\sigma$. Third, we uniformly randomly assigned each HR node to one of the seven communities keeping the number of nodes in each community the same as in the community structure of the original network. Fourth, we calculated $\sigma$ for each of the 10^5^ random assignments. Fifth, we calculated the average and standard deviation of $\sigma$ for the 10^5^ random assignments and then the Z score of $\sigma$ for the original networks.

The Z score for the wild and laboratory networks was equal to 0.66 (*p* = 0.51, uncorrected) and 2.51 (*p* = 0.024, Bonferroni corrected with multiplicity two), respectively. These results therefore statistically support the observation of concentration of HR nodes is in the laboratory network, and their non-concentration in the wild network.

**Supplementary Text S9. Statistical comparison of community structure**

We statistically compared different networks, specifically (i) wild *vs.* laboratory mouse networks where there were 237 wild mice and 21 laboratory mice; (ii) male *vs.* female wild mouse network, where there were 133 males and 104 females; (iii) young *vs*. old network, that compared 120 old mice, which we defined to be more than 8.5 weeks old, and 117 young mice, which were less than 8.5 weeks old; we selected the age threshold of 8.5 weeks to make the size of the two groups approximately equal, and (iv) comparison of networks of 71 mice from the HW site (a mixed arable and beef farm near Bristol, UK) and 166 mice from the other sites [1]; we selected the HW site because it was the single site where most wild mice were sampled. Our overall approach was to compare the actual networks with randomly generated ones, and from this we calculated the Z score for each of five similarity measures. There are many similarity measures that could be used to compare community structures [7] that, broadly, are pair counting methods, cluster matching methods, or information-theoretic methods [7–9]. We used five types of similarity measures, four of which are pair counting methods, and the fifth an information-theoretic method.

Specifically, the pair counting methods first classify all the possible $N\left( N-1 \right)/2$ pairs of nodes into four categories. We denote by $w_{11}$the number of node pairs that belong to the same community in both networks, by $w_{10}$ the number of node pairs that are in the same community in the first network but not in the second network, by $w_{01}$ the number of node pairs that are in the same community in the second network but not in the first network and by $w_{00}$ the number of node pairs that are not in the same community in either network. The total number of pairs of nodes is given by $w_{11}+w_{10}+w_{01}+w_{00}=N\left( N-1 \right)/2$. The four similarity measures based on pair-counting are as follows [7]: the Jaccard index $J=w_{11}/\left( w_{11}+w_{10}+w_{01} \right)$; the Rand similarity coefficient $R=2\left( w_{11}+w_{00} \right)/N\left( N-1 \right)$; the Fowlkes-Mallows similarity coefficient $FM=w_{11}/\sqrt{\left( w_{11}+w_{10} \right)\left( w_{11}+w_{01} \right)}$; the Minkowski coefficient $M=\sqrt{\left( w_{10}+w_{01} \right)/\left( w_{01}+w_{11} \right)}$.

The other similarity measure based on information theory is the variation of information (VI) [8], which is defined as follows. Denote the two node partitions to be compared by $X=\{X_{1},X_{2},\ldots,X_{k}\}$ and $Y=\{Y_{1},Y_{2},\ldots,Y_{l}\}$, where $k$ and $l$ are the number of communities in partition $X$ and $Y$ , respectively. The VI between the two partitions is defined as $\text{VI}\left( X,Y \right)=-\sum_{i,j} r_{ij}\left[ \log\left( r_{ij}/p_{i} \right)+\log\left( r_{ij}/q_{j} \right) \right]$, where $p_{i}=\left| X_{i} \right|/N$, $q_{j}=\left| Y_{j} \right|/N$ and $r_{ij}=\left| X_{i}\cap Y_{j} \right|/N$.

Similarity measures, including the ones described here, strongly depend on the number and size of communities [7]. It is therefore difficult to assert whether the value obtained for each similarity measure is large or small. To circumvent this problem, we calculated the similarity between two partitions relative to that obtained from random partitions. Given a similarity measure $S$ calculated from the original data, the Z score is defined as $Z_{S}=\left( S-\mu\right)/\sigma$, where $\mu$ and $\sigma$ are the average and standard deviation, respectively, of the same similarity measure calculated from random partitions. To avoid possible dependence of the Z value on the number of samples (*i.e*., number of mice) on which the correlation matrix is calculated, we generated random partitions as follows. As an example, for a given similarity measure, consider the comparison between the community structure of the wild male mouse network and that of the wild female mouse network. The male and female networks were derived from correlation matrices based on the 133 wild male mice and 104 wild female mice, respectively. First, we calculated the similarity measure between the community structure of the male network and that of the female network, giving $S$. Second, we combined males and females and drew 10 pairs of uniformly random networks maintaining the original number of mice in the two networks. In other words, in each randomly drawn pair of networks, one network was derived from 133 mice and the other network was derived from the remaining 104 mice. Third, for each of the randomly drawn pair of networks, we carried out the community detection. Fourth, we calculated the similarity measure between each pair of random networks. Finally, we calculated the Z score on the basis of the 10 random pairs of networks. It should be noted that the Z score of the Minkowski coefficient and the VI was multiplied by −1 because, unlike the other three similarity measures, more similar partitions yield smaller values of Minkowski coefficient or VI. Therefore, a negative Z value indicates that the pair of original networks (*e.g.*, male and female networks) is more different than are pairs of random networks. If the Z score is significantly smaller than zero, we conclude that, in this example, the male and female networks are significantly more different to each other compared to the difference between randomly generated pairs of networks.

We used an interval of 10^2^ MCMC sweeps in the first and second phases of the community detection to obtain the random pairs of networks used to calculate the Z score, and so to statistically compare the two networks using a certain number of MCMC sweeps. We used different numbers of MCMC sweeps for each comparison, and we determined this number as follows, which avoids excessively long computational time. First, for each network to be compared, we discarded transient sweeps before the partitions were sufficiently stable, where the number of MCMC sweeps to be discarded was determined using the same criterion as in **Supplementary Text S2**. Then, for each partition we ran a total of *T* = 10^3^ MCMC sweeps. Next, we computed the autocorrelation function $\chi\left( t \right)$ of the description length as a function of the number of sweeps $t$, which is given by

$$\begin{aligned} \chi\left( t \right)=\frac{1}{\left( T-t \right)\sigma_{H}^{2}}\sum_{t^{'}=1}^{T-t} \left[ H\left( t^{'} \right)-\left\langle H \right\rangle\right]\left[ H\left( t^{'}+t \right)-\left\langle H \right\rangle\right],\#\left( S5 \right) \end{aligned}$$

where $H\left( t \right)$ is the description length after $t$ sweeps; $\left\langle H \right\rangle$ and $\sigma_{H}$ are the average and the standard deviation of the description length, respectively. For each network, we averaged the autocorrelation function of the description length over 10^2^ realizations to obtain a smooth curve of the autocorrelation decay. The timescale at which the autocorrelation function decays towards zero is called the autocorrelation time $\tau$. It is expected that the autocorrelation function declines exponentially at large $t$ as $\chi\left( t \right)\sim e^{-t/\tau}$ [10]. Therefore, for each of the comparisons (*i.e*. wild *vs*. lab; sex; age; geographical site), we set the number of MCMC sweeps as two autocorrelation times $t=2\tau$.

The results of these comparisons are shown in **Supplementary Table S5**. This shows that the Z scores are below a 95 % significance level (*i.e*., |*Z*| *<* 1*.*96) in all cases except in two: (i) the wild *vs.* laboratory Rand coefficient’s Z score of −2.93, suggesting that the wild and laboratory networks are different and (ii) the site comparison with the VI similarity measure with a Z score of −2*.*04 suggesting that the networks between site HW and other sites are different. However, when correcting for the multiple comparisons that we have made, these Z scores do not remain significant. Therefore, formally we conclude that we cannot detect differences in the immune network structure between wild and laboratory, young and old, male and female mice, or mice from different geographical sites.

**
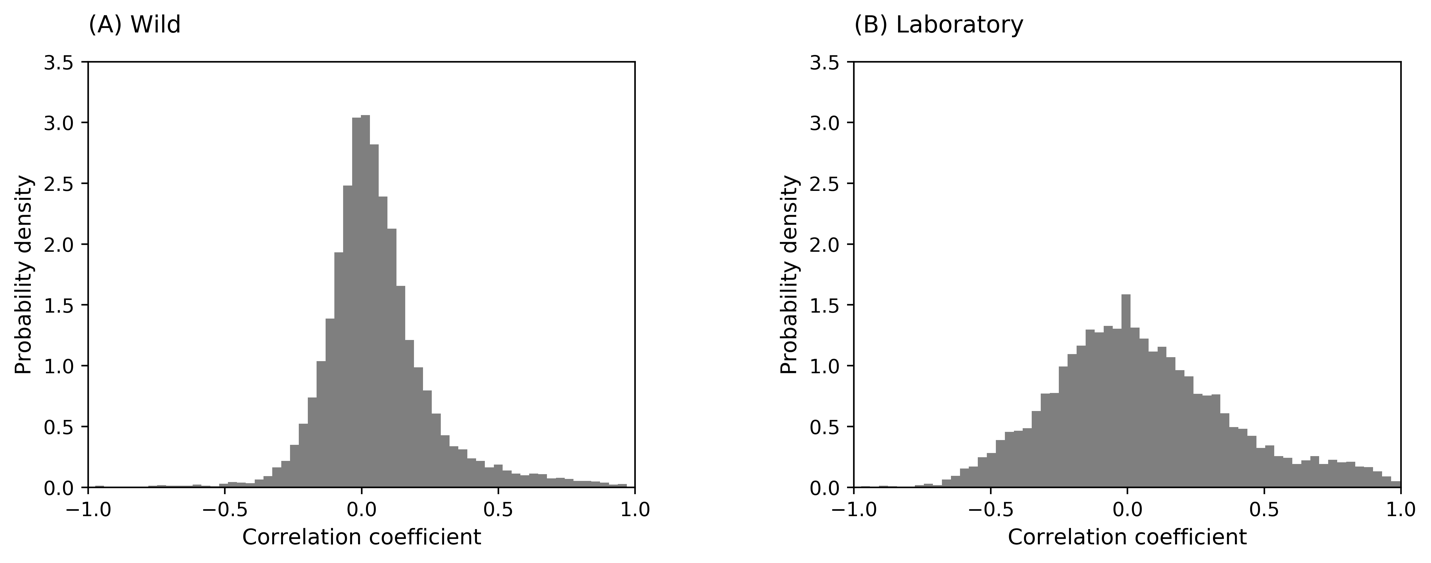
**

**Supplementary Figure S1.** Probability density function of correlation coefficients for the (A) wild and (B) laboratory mice.


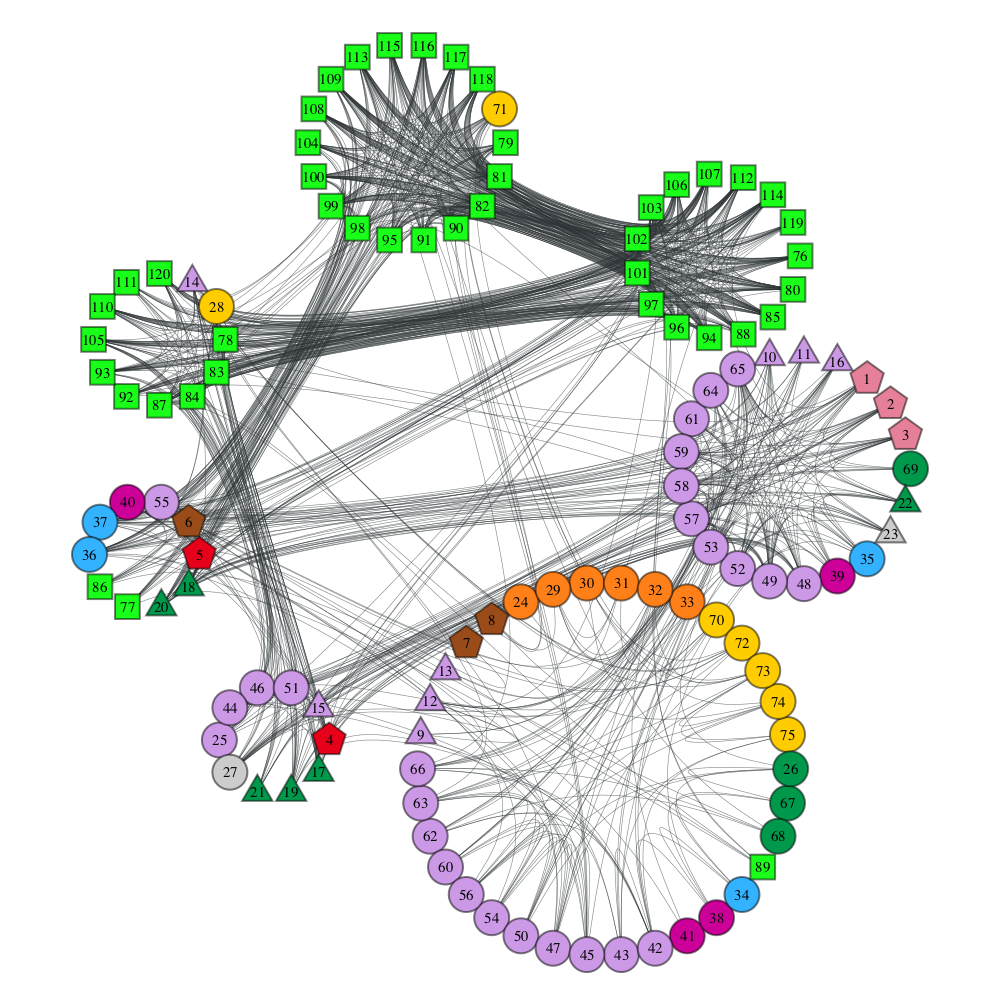


**Supplementary Figure S2.** Wild mouse network with a threshold of 0.2. The nodes are labelled according to **Supplementary Table S6**.


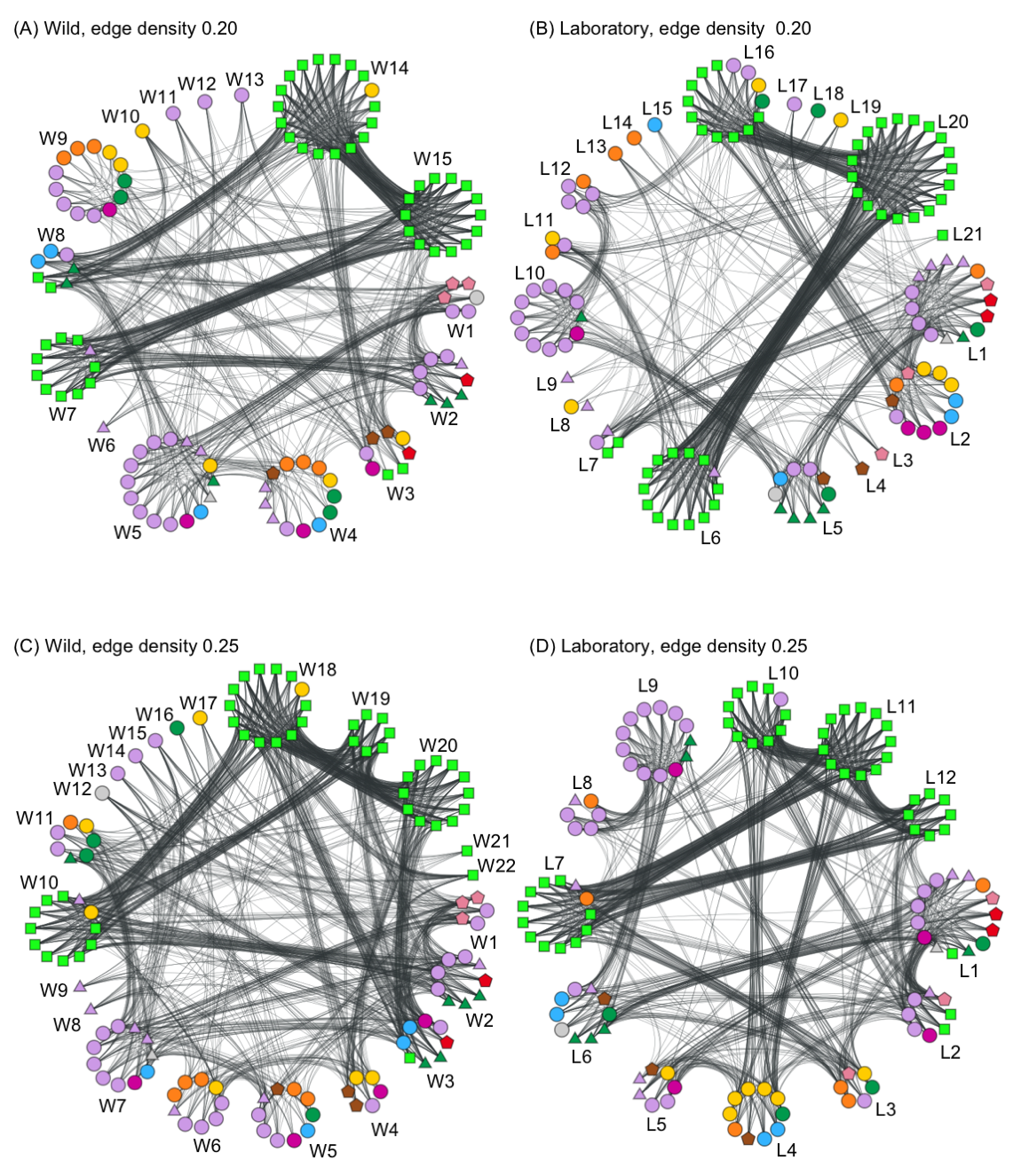


**Supplementary Figure S3.** Networks with lower thresholds. (A) Wild and (B) laboratory networks with thresholds of 0.17 and 0.31, respectively yielding an edge density of 0.20. These two are similar in that they consist of networks of 10 communities (and a number of nodes that do not belong to any community) and that most of the CR nodes are in three communities. (C) Wild and (D) laboratory networks with thresholds of 0.14 and 0.24, respectively, yielding an edge density of 0.25. These two are similar in that they consist of networks of 12 communities (with the wild network also having a number of nodes that do not belong to any community) and that 42 of the 45 CR nodes are in four communities.

**Supplementary Figure S4.** Th1 *vs.* Th2 polarization in the wild mice network, showing only the edges that derive from the (A) IFN-γ nodes, (B) IL-4 nodes and (C) IL-13 nodes, which are shown with red halos in each case. The nodes are labelled according to **Supplementary Table S6**.


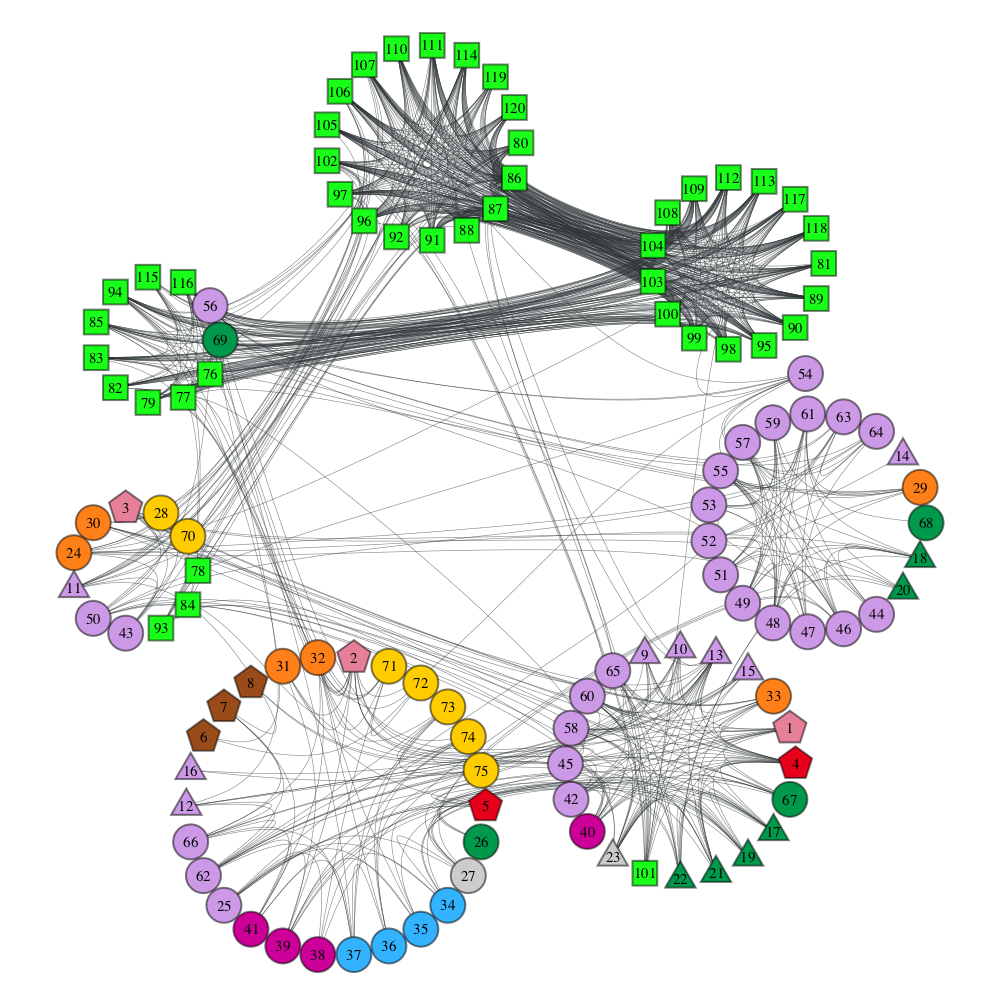


**Supplementary Figure S5.** Laboratory mouse network with a threshold of 0.42. The nodes are labelled according to **Supplementary Table S6**.

**
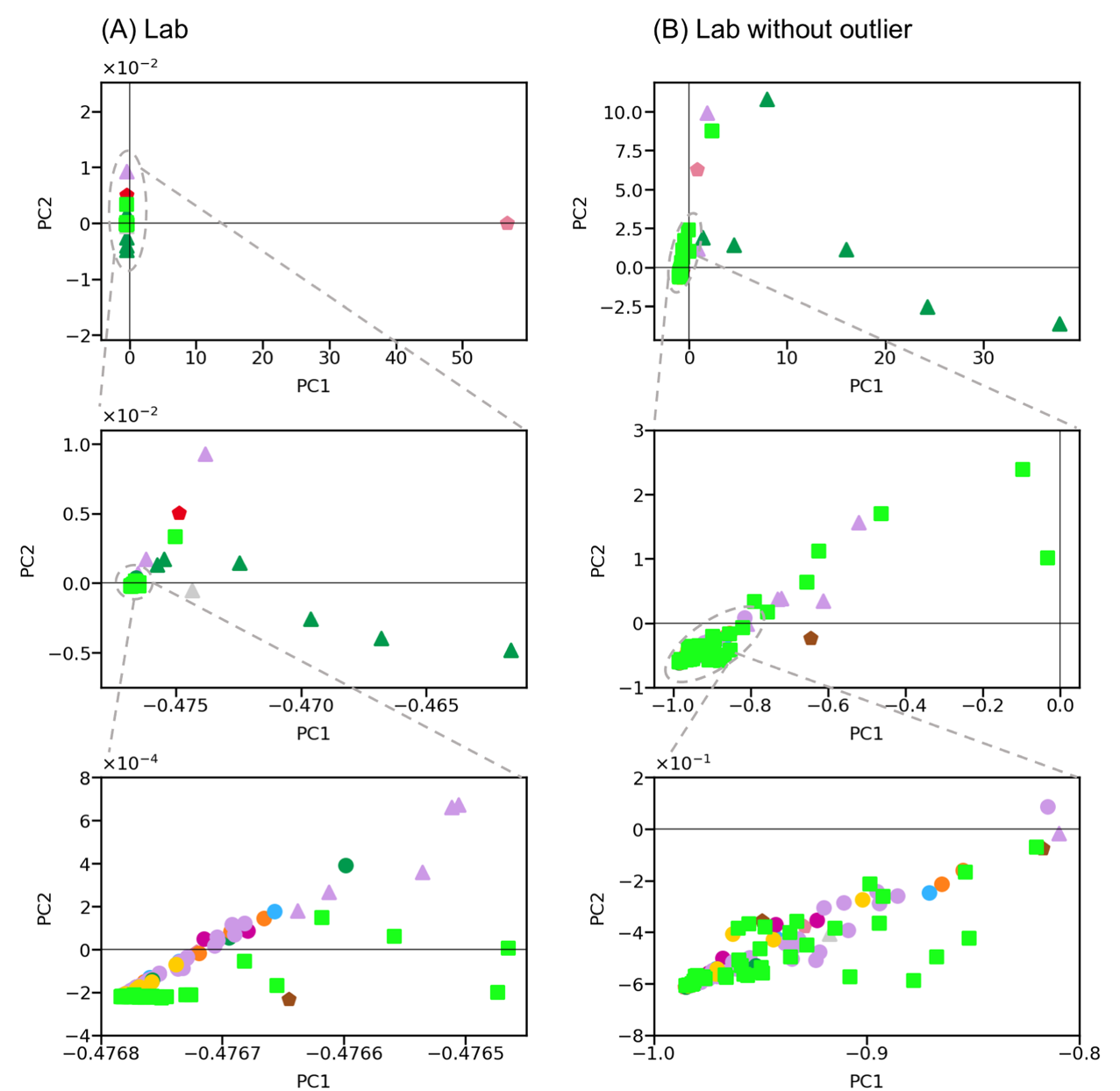
**

**Supplementary Figure S6.** PCA for the laboratory mouse data. Projection of the first (PC1) and the second (PC2) principal components of the immune measures for laboratory mice (A) with and (B) without the outlier (*i.e.*, number of spleen cells), with three levels of expansion (shown by the dotted lines). See **Fig. 1A** for the shape and colour of the nodes.


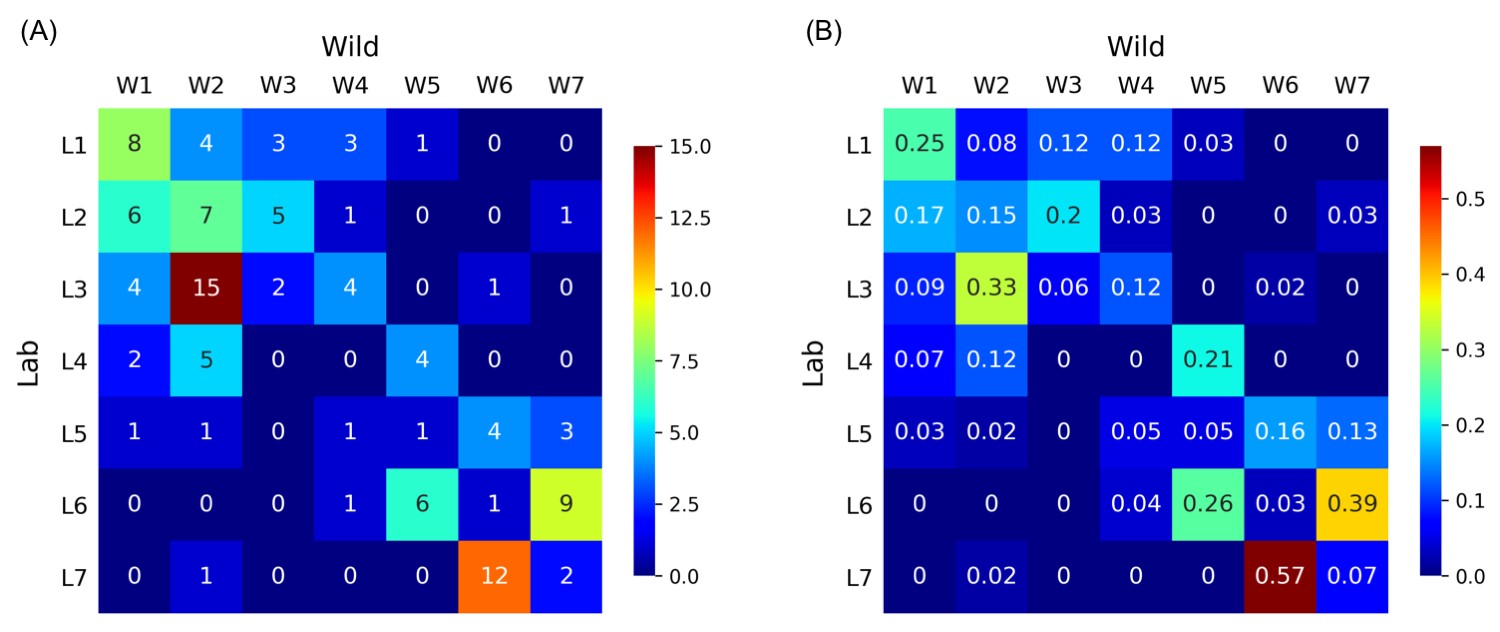


**Supplementary Figure S7.** Similarity between communities of the wild and laboratory mouse networks, as the (A) number of common nodes and (B) Jaccard index.


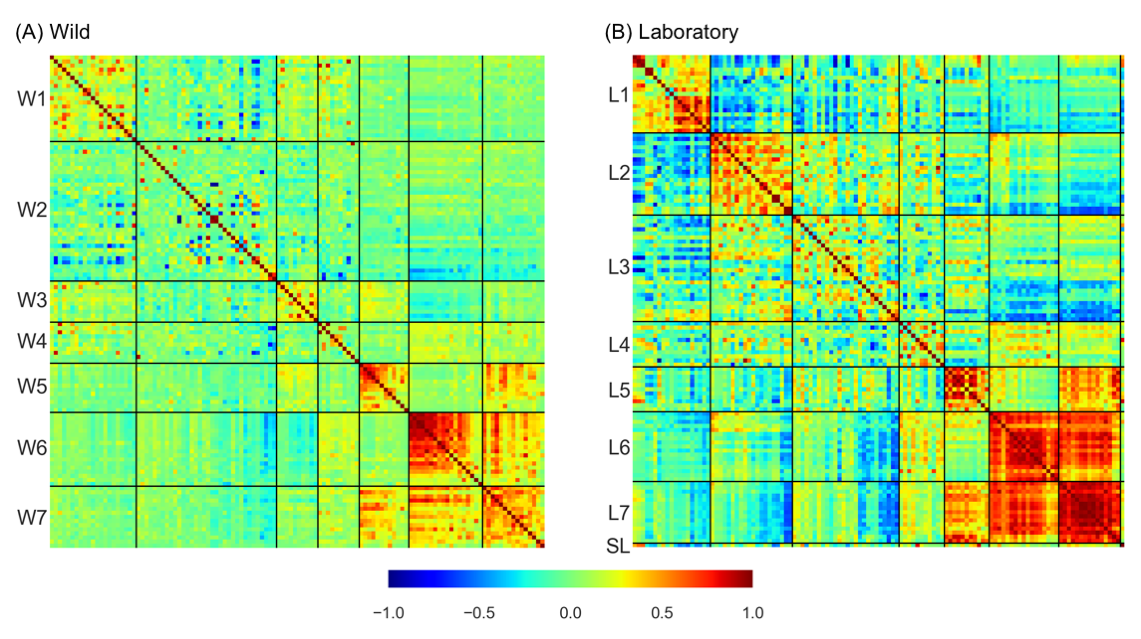


**Supplementary Figure S8.** Correlation matrices arranged by community blocks for (A) wild and (B) laboratory mice.


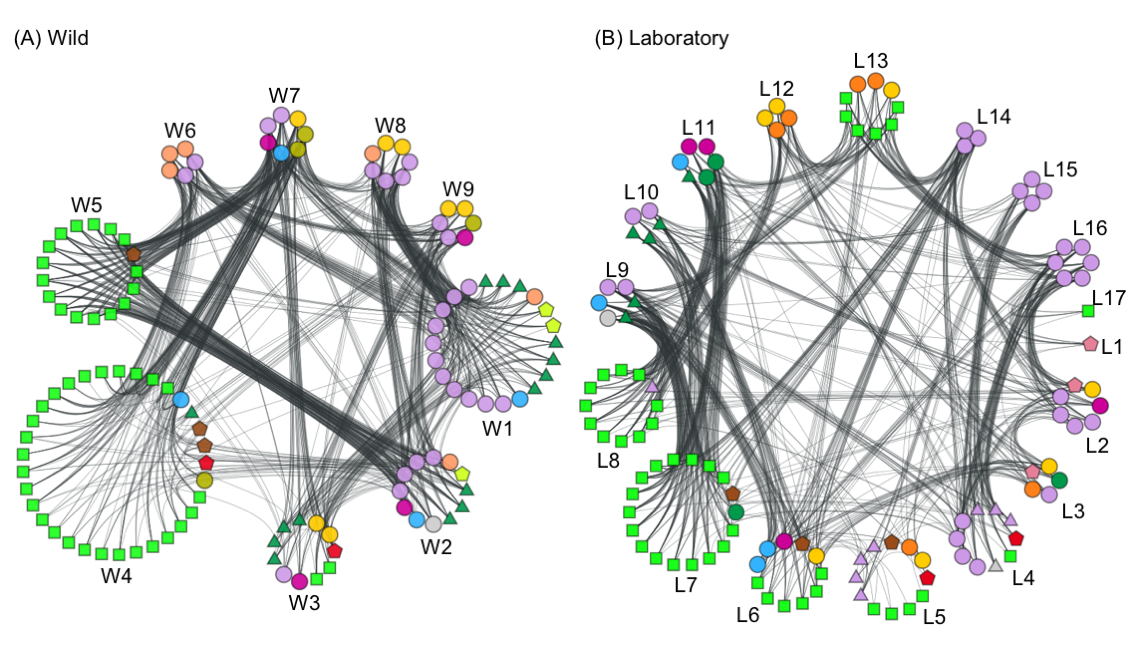


**Supplementary Figure S9.** Negative networks for (A) wild and (B) laboratory mice. In each panel, we used the smallest negative threshold that yielded a connected negative network.

**Supplementary Table S1.** List of the MFI, FACS, and Cytokine Responses used as nodes of the immune response networks. For CR each of the five culture conditions are shown first, followed by each of the nine cytokines [1].

| **Category** | **Measures** | | |
| --- | --- | --- | --- |
| Mean fluorescence index (MFI) | CD11b- CD27- MFI CD69 (S1) | CD11b- CD27 + MFI KLRG1 (S2) | IgD-/CD38+ GL7- MFI PNA |
|  | CD11b- CD27 + MFI CD69 (S2) | CD11b+ CD27 + MFI KLRG1 (S3) | IgD-/CD38+ GL7- MFI MHC-II |
|  | CD11b+ CD27 + MFI CD69 (S3) | CD11b+ CD27- MFI KLRG1 (S4) | IgD-/CD38Lo GL7Hi MFI PNA |
|  | CD11b+ CD27- MFI CD69 (S4) | IgD+ MFI PNA | IgD-/CD38Lo GL7Hi MFI MHC-II |
|  | CD11b- CD27- MFI KLRG1 (S1) | IgD+ MFI MHC-II | MFI MHC-II of DCs |
| Fluorescence activated cell sorted (FACS) | % CD3+ (T cells) | CD27- CD11b- of NKp46+ (S1) | % D-G2Hi-G2Lo+H- |
|  | % NKp46+ CD3- (NK cells) | CD27+ CD11b- of NKp46+ (S2) | % D-G2Hi-G2Lo-H+ |
|  | % CD19+ (B cells) | CD27+ CD11b+ of NKp46+ (S3) | % D-G2Hi-G2Lo-H- |
|  | % CD11c+ CD3- (DCs) | CD27-CD11b+ of NKp46+ (S4) | % D+G2- |
|  | % CD11b+ (Myeloid) | % KLRG1+ of NKp46+ (TD NKs) | % D+G2+ |
|  | % CD4- CD8+ | Ly49D+ of NKp46+ (Act.) | % D-G2+ |
|  | % CD4+ CD8- | Ly49H+ of NKp46+ (Act.) | % D-G2- |
|  | CD4+ CD8+ | % Ly49G2Hi | % IgD+ (naïve) |
|  | CD4- CD8- | % Ly49G2Lo | % CD38+ GL7- of 19 |
|  | CD4+/CD8+ ratio | % D+G2Hi+G2Lo-H+ | % CD38Lo GL7Hi of CD19 |
|  | % CD62L+ CD44- Naïve CD8 | % D+G2Hi+G2Lo-H- | M1 Resident Macrophages (F4/80+ Ly6G-) |
|  | % CD62L+ CD44+ Int. CD8 | % D+G2Hi-G2Lo+H+ | M2 Monocytes (F4/80+ Ly6G lo) |
|  | % CD62L- CD44+ Effector CD8 | % D+G2Hi-G2Lo+H- | M3 Hyper-granulocytic myeloid cells (F4/80+ Ly6G int.) |
|  | % KLRG-1+ of CD8+ | % D+G2Hi-G2Lo-H+ |  |
|  | % CD62L+ CD44- Naïve CD4 | % D+G2Hi-G2Lo-H- | M4 Polymorphonuclear cells (F4/80 variable Ly6G Hi) |
|  | % CD62L+ CD44+ Int. CD4 | % D-G2Hi+G2Lo-H+ |  |
|  | % CD62L- CD44+ Effector CD4 | % D-G2Hi+G2Lo-H- | M5 Neutrophils (FSC-lo PMNs) |
|  | % CD25+ FoxP3+ (Treg) | % D-G2Hi-G2Lo+H+ | M6 MDSC (FSC-Hi PMNs) |
| Cytokine Response (CR) | RPMI IL-1β | CD3/CD28 IL-13 | LPS IL-10 |
|  | RPMI IL-4 | CD3/CD28 IFN-γ | LPS IL-12p40 |
|  | RPMI IL-6 | CD3/CD28 MIP-2α | LPS IL-12p70 |
|  | RPMI IL-10 | CPG IL-1β | LPS IL-13 |
|  | RPMI IL-12p40 | CPG IL-4 | LPS IFN-γ |
|  | RPMI IL-12p70 | CPG IL-6 | LPS MIP-2α |
|  | RPMI IL-13 | CPG IL-10 | PG IL-1β |
|  | RPMI IFN-γ | CPG IL-12p40 | PG IL-4 |
|  | RPMI MIP-2α | CPG IL-12p70 | PG IL-6 |
|  | CD3/CD28 IL-1β | CPG IL-13 | PG IL-10 |
|  | CD3/CD28 IL-4 | CPG IFN-γ | PG IL-12p40 |
|  | CD3/CD28 IL-6 | CPG MIP-2α | PG IL-12p70 |
|  | CD3/CD28 IL-10 | LPS IL-1β | PG IL-13 |
|  | CD3/CD28 IL-12p40 | LPS IL-4 | PG IFN-γ |
|  | CD3/CD28 IL-12p70 | LPS IL-6 | PG MIP-2α |

**Supplementary Table S2.** Distribution of CR nodes within communities, showing the number of CR nodes in each community of wild mice (W) and laboratory mice (L) in terms of the five types of culture conditions (top) and the nine types of cytokines (bottom) [1].

|  | **Wild mouse communities** | | | | | | |  | **Laboratory mouse communities** | | | | | | | |
| --- | --- | --- | --- | --- | --- | --- | --- | --- | --- | --- | --- | --- | --- | --- | --- | --- |
|  | **W1** | **W2** | **W3** | **W4** | **W5** | **W6** | **W7** |  | **L1** | **L2** | **L3** | **L4** | **L5** | **L6** | **L7** | **SL** |
| **RPMI** |  |  |  | 1 | 3 | 3 | 2 |  |  |  |  | 2 | 5 | 1 | 1 |  |
| **CD3/CD28** |  |  | 1 | 1 | 3 | 2 | 2 |  |  |  |  | 1 | 1 | 5 | 2 |  |
| **LPS** |  |  |  |  |  | 4 | 5 |  |  | 1 |  |  | 1 | 3 | 4 |  |
| **CpG** |  |  |  |  | 3 | 3 | 3 |  |  |  |  |  |  | 5 | 4 |  |
| **PG** |  |  |  |  | 1 | 5 | 3 |  |  |  |  |  | 2 | 3 | 4 |  |
| **IL-1𝛽** |  |  |  |  |  |  | 5 |  |  |  |  |  | 3 |  | 2 |  |
| **IL-4** |  |  |  | 2 |  | 3 |  |  |  |  |  |  | 1 | 1 | 3 |  |
| **IL-6** |  |  |  |  | 3 |  | 2 |  |  |  |  | 1 |  | 4 |  |  |
| **IL-10** |  |  |  |  |  | 2 | 3 |  |  |  |  |  | 2 | 3 |  |  |
| **IL-12p70** |  |  |  |  |  | 5 |  |  |  |  |  |  |  |  | 5 |  |
| **IL-12p40** |  |  |  |  |  | 2 | 2 |  |  |  |  |  | 1 | 2 | 2 |  |
| **IL-13** |  |  |  |  |  | 5 |  |  |  |  |  |  | 1 | 1 | 3 |  |
| **IFN-𝛾** |  |  |  |  | 3 |  | 2 |  |  | 1 |  |  | 1 | 3 |  |  |
| **MIP-2𝛼** |  |  |  |  | 4 |  | 1 |  |  |  |  | 2 |  | 3 |  |  |

**Supplementary Table S3.** Mean and standard deviation of the number of common nodes and the Jaccard index between each CR community in the random shuffled wild network and each CR community in the random shuffled laboratory network.

|  | **Number of common nodes** | | | |  | |  | | **Jaccard index** | | | |
| --- | --- | --- | --- | --- | --- | --- | --- | --- | --- | --- | --- | --- |
|  | **W5'** | **W6'** | **W7'** |  | |  | | **W5'** | | **W6'** | **W7'** |  |
| **L5'** | 2.8 ± 1.3 | 4.0 ± 1.4 | 3.5 ± 1.4 |  | | **L5'** | | 0.14 ± 0.07 | | 0.17 ± 0.07 | 0.15 ± 0.07 |  |
| **L6'** | 4.2 ± 1.5 | 6.0 ± 1.6 | 5.3 ± 1.6 |  | | **L6'** | | 0.17 ± 0.07 | | 0.21 ± 0.07 | 0.20 ± 0.07 |  |
| **L7'** | 3.5 ± 1.4 | 5.0 ± 1.6 | 4.4 ± 1.5 |  | | **L7'** | | 0.15 ± 0.07 | | 0.19 ± 0.07 | 0.18 ± 0.07 |  |

**Supplementary Table S4.** Z scores of the number of common nodes and the Jaccard index between each CR community in the wild network and in the laboratory network, relative to the CR community structure obtained by random shuffling of the nodes.

|  | **Number of**  **common nodes** | | |  |  | **Jaccard index** | | |
| --- | --- | --- | --- | --- | --- | --- | --- | --- |
|  | **W5** | **W6** | **W7** |  |  | **W5** | **W6** | **W7** |
| **L5** | −1.41 | −0.17 | −0.38 |  | **L5** | −1.20 | −0.10 | −0.32 |
| **L6** | 1.38 | −3.07 | 2.57 |  | **L6** | 1.38 | −2.63 | 2.88 |
| **L7** | −2.54 | 4.26 | −1.62 |  | **L7** | −2.15 | 5.45 | −1.50 |

**Supplementary Table S5.** Similarity of the community structure of pairs of networks shown as the Z score. The similarity measures used are the Jaccard index, Rand coefficient, Fowlkes-Mallows coefficient (FM), Minkowski coefficient and the variation of information (VI).

|  | **Jaccard** | **Rand** | **FM** | **Minkowski** | **VI** |
| --- | --- | --- | --- | --- | --- |
| **Wild *vs*. laboratory** | 0.24 | −2.93 | 0.16 | 1.34 | 0.19 |
| **Male *vs*. female** | −0.37 | −0.09 | −0.44 | −0.48 | −1.20 |
| **Young *vs*. old** | −0.96 | 1.16 | −1.05 | −0.86 | −0.89 |
| **HW *vs*. other sites** | −1.42 | 0.59 | −1.73 | −0.85 | −2.04 |

**Supplementary Table S6.** Numerical label of the nodes used in **Supplementary Figs. S2, S4** and **S5**.

| **Node** | **Immune measure** | **Category** |
| --- | --- | --- |
| 1 | Mass (g) | Body measures |
| 2 | Spleen mass (g) | Body measures |
| 3 | Number of Spleen Cells | Body measures |
| 4 | IgG | Antibodies |
| 5 | IgE | Antibodies |
| 6 | SAP | Serum proteins |
| 7 | Haptoglobin | Serum proteins |
| 8 | AAT | Serum proteins |
| 9 | CD11b- CD27- MFI CD69 (S1) | MFI NK cells |
| 10 | CD11b- CD27 + MFI CD69 (S2) | MFI NK cells |
| 11 | CD11b+ CD27 + MFI CD69 (S3) | MFI NK cells |
| 12 | CD11b+ CD27- MFI CD69 (S4) | MFI NK cells |
| 13 | CD11b- CD27- MFI KLRG1 (S1) | MFI NK cells |
| 14 | CD11b- CD27 + MFI KLRG1 (S2) | MFI NK cells |
| 15 | CD11b+ CD27 + MFI KLRG1 (S3) | MFI NK cells |
| 16 | CD11b+ CD27- MFI KLRG1 (S4) | MFI NK cells |
| 17 | IgD+ MFI PNA | MFI B cells |
| 18 | IgD+ MFI MHC-II | MFI B cells |
| 19 | IgD-/CD38+ GL7- MFI PNA | MFI B cells |
| 20 | IgD-/CD38+ GL7- MFI MHC-II | MFI B cells |
| 21 | IgD-/CD38Lo GL7Hi MFI PNA | MFI B cells |
| 22 | IgD-/CD38Lo GL7Hi MFI MHC-II | MFI B cells |
| 23 | MFI MHC-II of DCs | MFI dendritic cells |
| 24 | % CD3+ (T cells) | FACS T cells |
| 25 | % NKp46+ CD3- (NK cells) | FACS NK cells |
| 26 | % CD19+ (B cells) | FACS B cells |
| 27 | % CD11c+ CD3- (DCs) | FACS dendritic cells |
| 28 | % CD11b+ (Myeloid) | FACS Myeloid |
| 29 | % CD4- CD8+ | FACS T cells |
| 30 | % CD4+ CD8- | FACS T cells |
| 31 | CD4+ CD8+ | FACS T cells |
| 32 | CD4- CD8- | FACS T cells |
| 33 | CD4+/CD8+ ratio | FACS T cells |
| 34 | % CD62L+ CD44- Naïve CD8 | FACS CD8^+^ T cells |
| 35 | % CD62L+ CD44+ Int. CD8 | FACS CD8^+^ T cells |
| 36 | % CD62L- CD44+ Effector CD8 | FACS CD8^+^ T cells |
| 37 | % KLRG-1+ of CD8+ | FACS CD8^+^ T cells |
| 38 | % CD62L+ CD44- Naïve CD4 | FACS CD4^+^ T cells |
| 39 | % CD62L+ CD44+ Int. CD4 | FACS CD4^+^ T cells |
| 40 | % CD62L- CD44+ Effector CD4 | FACS CD4^+^ T cells |
| 41 | % CD25+ FoxP3+ (Treg) | FACS CD4^+^ T cells |
| 42 | CD27- CD11b- of NKp46+ (S1) | FACS NK cells |
| 43 | CD27+ CD11b- of NKp46+ (S2) | FACS NK cells |
| 44 | CD27+ CD11b+ of NKp46+ (S3) | FACS NK cells |
| 45 | CD27-CD11b+ of NKp46+ (S4) | FACS NK cells |
| 46 | % KLRG1+ of NKp46+ (TD NKs) | FACS NK cells |
| 47 | Ly49D+ of NKp46+ (Act.) | FACS NK cells |
| 48 | Ly49H+ of NKp46+ (Act.) | FACS NK cells |
| 49 | % Ly49G2Hi | FACS NK cells |
| 50 | % Ly49G2Lo | FACS NK cells |
| 51 | % D+G2Hi+G2Lo-H+ | FACS NK cells |
| 52 | % D+G2Hi+G2Lo-H- | FACS NK cells |
| 53 | % D+G2Hi-G2Lo+H+ | FACS NK cells |
| 54 | % D+G2Hi-G2Lo+H- | FACS NK cells |
| 55 | % D+G2Hi-G2Lo-H+ | FACS NK cells |
| 56 | % D+G2Hi-G2Lo-H- | FACS NK cells |
| 57 | % D-G2Hi+G2Lo-H+ | FACS NK cells |
| 58 | % D-G2Hi+G2Lo-H- | FACS NK cells |
| 59 | % D-G2Hi-G2Lo+H+ | FACS NK cells |
| 60 | % D-G2Hi-G2Lo+H- | FACS NK cells |
| 61 | % D-G2Hi-G2Lo-H+ | FACS NK cells |
| 62 | % D-G2Hi-G2Lo-H- | FACS NK cells |
| 63 | % D+G2- | FACS NK cells |
| 64 | % D+G2+ | FACS NK cells |
| 65 | % D-G2+ | FACS NK cells |
| 66 | % D-G2- | FACS NK cells |
| 67 | % IgD+ (naïve) | FACS B cells |
| 68 | % CD38+ GL7- of 19 | FACS B cells |
| 69 | % CD38Lo GL7Hi of CD19 | FACS B cells |
| 70 | M1 Resident Macrophages (F4/80+ Ly6G-) | FACS Myeloid |
| 71 | M2 Monocytes (F4/80+ Ly6G lo) | FACS Myeloid |
| 72 | M3 Hyper-granulocytic myeloid cells (F4/80+ Ly6G int.) | FACS Myeloid |
| 73 | M4 Polymorphonuclear cells (F4/80 variable Ly6G Hi) | FACS Myeloid |
| 74 | M5 Neutrophils (FSC-lo PMNs) | FACS Myeloid |
| 75 | M6 MDSC (FSC-Hi PMNs) | FACS Myeloid |
| 76 | IL-1beta RPMI | CR |
| 77 | IL-4 RPMI | CR |
| 78 | IL-6 RPMI | CR |
| 79 | IL-10 RPMI | CR |
| 80 | IL-12p40 RPMI | CR |
| 81 | IL-12p70 RPMI | CR |
| 82 | IL-13 RPMI | CR |
| 83 | IFN-gamma RPMI | CR |
| 84 | MIP-2alpha RPMI | CR |
| 85 | IL-1beta CD3/CD28 | CR |
| 86 | IL-4 CD3/CD28 | CR |
| 87 | IL-6 CD3/CD28 | CR |
| 88 | IL-10 CD3/CD28 | CR |
| 89 | IL-12p40 CD3/CD28 | CR |
| 90 | IL-12p70 CD3/CD28 | CR |
| 91 | IL-13 CD3/CD28 | CR |
| 92 | IFN-gamma CD3/CD28 | CR |
| 93 | MIP-2alpha CD3/CD28 | CR |
| 94 | IL-1beta LPS | CR |
| 95 | IL-4 LPS | CR |
| 96 | IL-6 LPS | CR |
| 97 | IL-10 LPS | CR |
| 98 | IL-12p40 LPS | CR |
| 99 | IL-12p70 LPS | CR |
| 100 | IL-13 LPS | CR |
| 101 | IFN-gamma LPS | CR |
| 102 | MIP-2alpha LPS | CR |
| 103 | IL-1beta CPG | CR |
| 104 | IL-4 CPG | CR |
| 105 | IL-6 CPG | CR |
| 106 | IL-10 CPG | CR |
| 107 | IL-12p40 CPG | CR |
| 108 | IL-12p70 CPG | CR |
| 109 | IL-13 CPG | CR |
| 110 | IFN-gamma CPG | CR |
| 111 | MIP-2alpha CPG | CR |
| 112 | IL-1beta PG | CR |
| 113 | IL-4 PG | CR |
| 114 | IL-6 PG | CR |
| 115 | IL-10 PG | CR |
| 116 | IL-12p40 PG | CR |
| 117 | IL-12p70 PG | CR |
| 118 | IL-13 PG | CR |
| 119 | IFN-gamma PG | CR |
| 120 | MIP-2alpha PG | CR |

**References**

1. S. Abolins, E. King, L. Lazarou, L. Weldon, L. Hughes, P. Drescher, J. Raynes, J. Hafalla, M. E. Viney, and E. M. Riley. The comparative immunology of wild and laboratory mice, *Mus musculus domesticus*. Nat. Commun., 8:14811, 2017.
2. T. P. Peixoto. Bayesian stochastic blockmodeling. In: P. Doreian, V. Batagelj, and A. Ferligoj, editors. Advances in Network Clustering and Blockmodeling. Jon Wiley & Sons, Ltd.: Oxford, UK, 2020.
3. T. Peixoto. Efficient Monte Carlo and greedy heuristic for the inference of stochastic block models. Phys. Rev. E, 89:012804, 2014.
4. T. Peixoto. The graph-tool python library [internet]. Figshare, 2014 [updated 2019 Jul 13, cited 2020 Feb 10]. Available from: [https://graph-tool.skewed.de.](https://graph-tool.skewed.de/)
5. N. Metropolis, A. W. Rosenbluth, M. N. Rosenbluth, A. H. Teller, and E. Teller. Equation of state calculations by fast computing machines. J. Chem. Phys., 21:1087, 1953.
6. K. Hastings, W. Monte Carlo sampling methods using Markov chains and their applications. Biometrika, 57:97, 1970.
7. A. L. Traud, E. D. Kelsic, P. J. Mucha, and M. A. Porter. Comparing community structure to characteristics in online collegiate social networks. SIAM Rev., 53:526, 2011.
8. M. Meilă. Comparing clusterings—an information-based distance. J. Multivar. Anal., 98:873, 2007.
9. B. Karrer, E. Levina, and M. E. J. Newman. Robustness of community structure in networks. Phys. Rev. E, 77:046119, 2008.
10. M. Newman and G. Barkema. Monte Carlo Methods in Statistical Physics. Oxford University Press: New York, USA, 1999.
11. B. K. Fosdick, D. B. Larremore, J. Nishimura, and J. Ugander. Configuring random graph models with fixed degree sequences. SIAM Rev., 60:315, 2018.
